# Correction: The modeling method for vibration characteristics analysis of composite laminated rotationally stiffened shell

**DOI:** 10.1371/journal.pone.0313986

**Published:** 2024-11-13

**Authors:** The PLOS ONE Staff

## Notice of Republication

This article was republished on November 6, 2024, to correct an error in affiliation 1 that was introduced during the typesetting process. The publisher apologizes for this error. Please download this article again to view the corrected version. Both the originally published, uncorrected article and the republished, corrected article are provided here for reference.

## Supporting information

S1 File Originally published, uncorrected article. (PDF)

S2 File Republished, corrected article. (PDF)

## References

[1] Shi D, Zhang H, Ding Y, Yang C, Cheng T (2024) The modeling method for vibration characteristics analysis of composite laminated rotationally stiffened shell. PLoS ONE 19(6): e0299586. 10.1371/journal.pone.0299586 38889193 PMC11185495

