# Supplementary figures and images for: The modeling method for vibration characteristics analysis of composite laminated rotationally stiffened shell
Source: PLoS One. 2024 Jun 18;19(6):e0299586. doi: 10.1371/journal.pone.0299586 (PMC11185495; doi:10.1371/journal.pone.0299586)

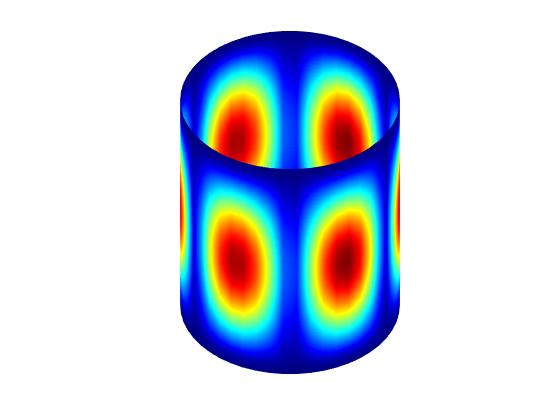

Supplement: S1 File — (ZIP) [file pone.0299586.s001.zip › supporting information/Fig 11(1).jpg]

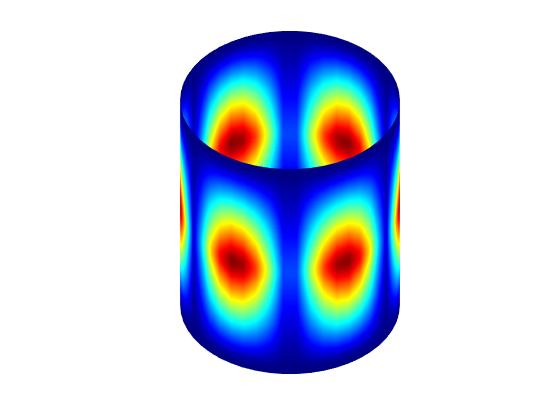

Supplement: S1 File — (ZIP) [file pone.0299586.s001.zip › supporting information/Fig 11(2).jpg]

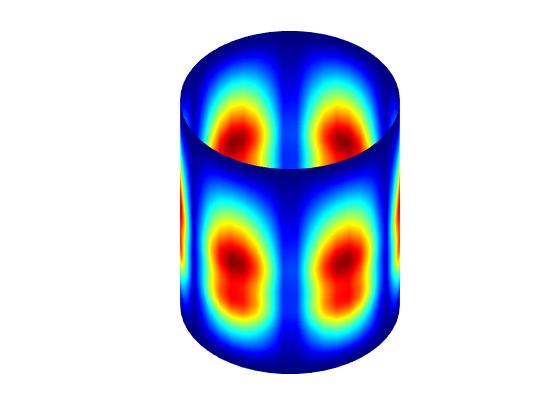

Supplement: S1 File — (ZIP) [file pone.0299586.s001.zip › supporting information/Fig 11(3).jpg]

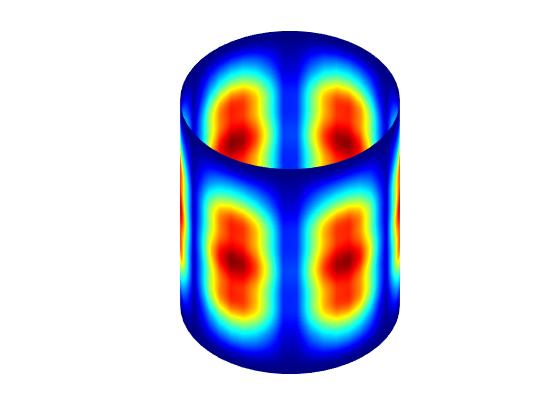

Supplement: S1 File — (ZIP) [file pone.0299586.s001.zip › supporting information/Fig 11(4).jpg]

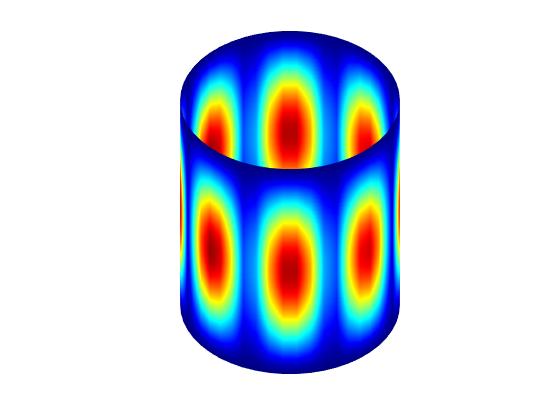

Supplement: S1 File — (ZIP) [file pone.0299586.s001.zip › supporting information/Fig 11(5).jpg]

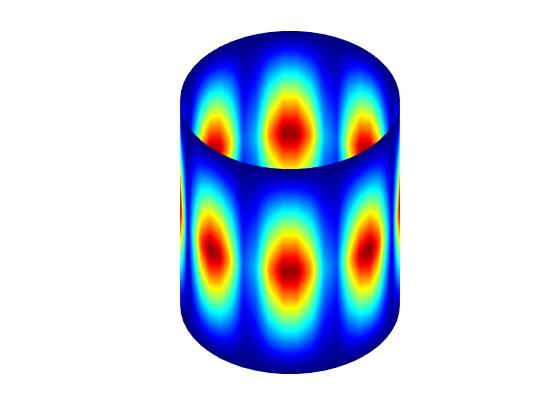

Supplement: S1 File — (ZIP) [file pone.0299586.s001.zip › supporting information/Fig 11(6).jpg]

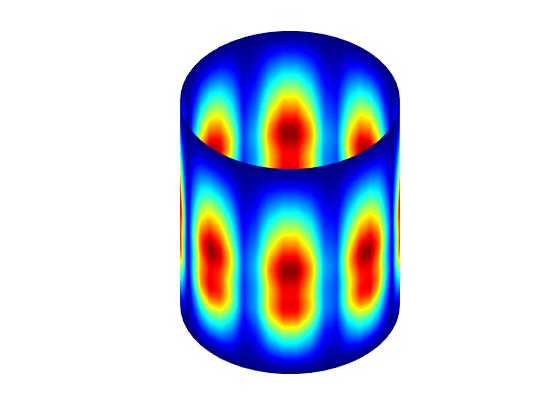

Supplement: S1 File — (ZIP) [file pone.0299586.s001.zip › supporting information/Fig 11(7).jpg]

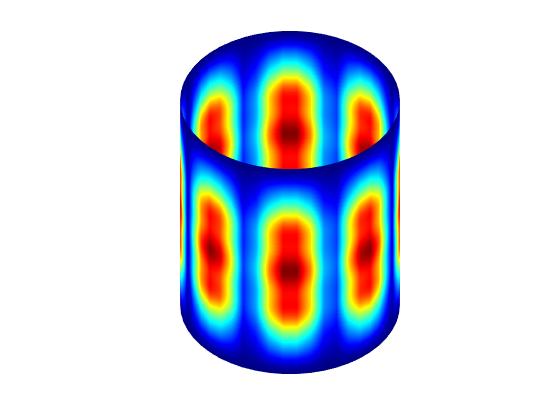

Supplement: S1 File — (ZIP) [file pone.0299586.s001.zip › supporting information/Fig 11(8).jpg]

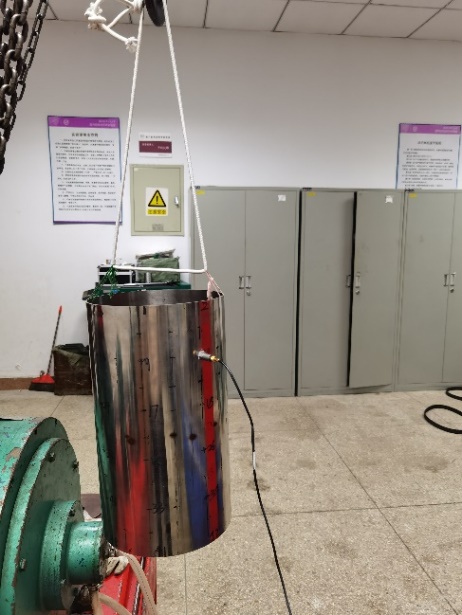

Supplement: S1 File — (ZIP) [file pone.0299586.s001.zip › supporting information/Fig 13(1).jpg]

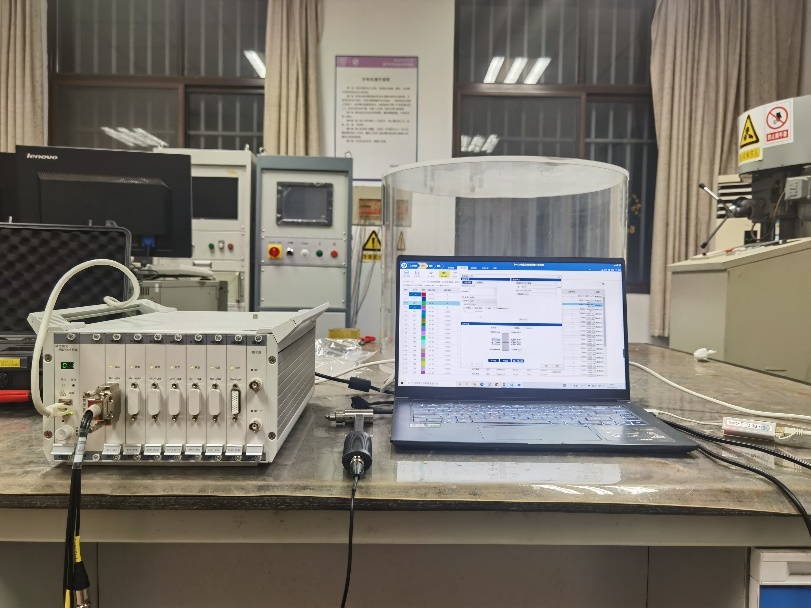

Supplement: S1 File — (ZIP) [file pone.0299586.s001.zip › supporting information/Fig 13(2).jpg]

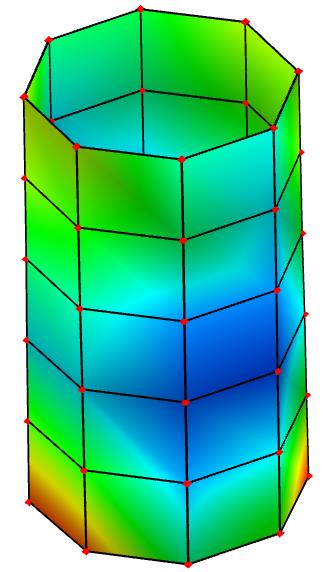

Supplement: S1 File — (ZIP) [file pone.0299586.s001.zip › supporting information/Fig 14(1).jpg]

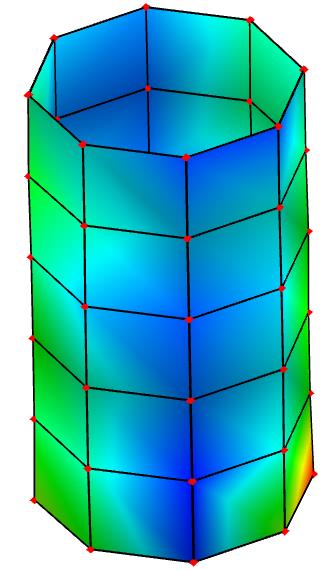

Supplement: S1 File — (ZIP) [file pone.0299586.s001.zip › supporting information/Fig 14(2).jpg]

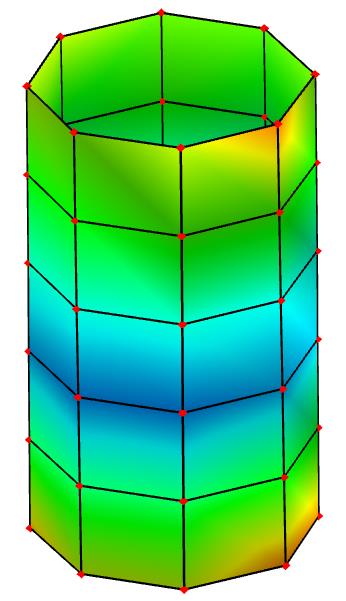

Supplement: S1 File — (ZIP) [file pone.0299586.s001.zip › supporting information/Fig 14(3).jpg]

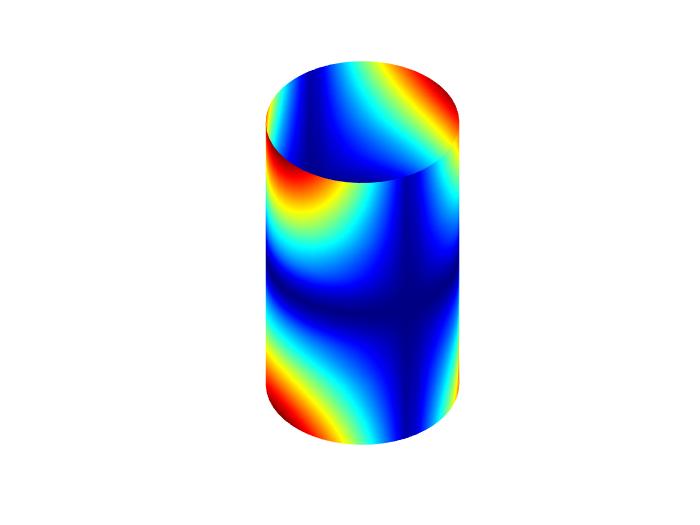

Supplement: S1 File — (ZIP) [file pone.0299586.s001.zip › supporting information/Fig 14(4).jpg]

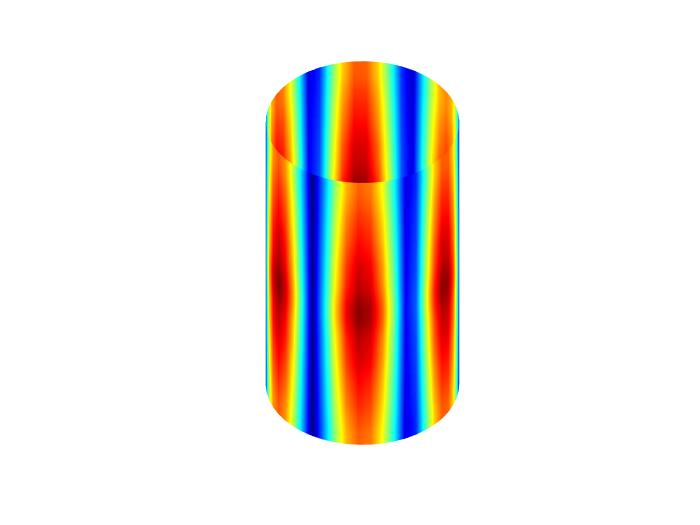

Supplement: S1 File — (ZIP) [file pone.0299586.s001.zip › supporting information/Fig 14(5).jpg]

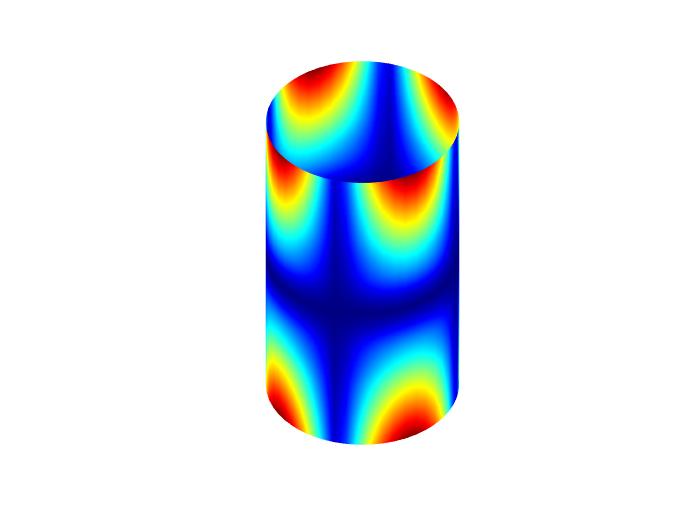

Supplement: S1 File — (ZIP) [file pone.0299586.s001.zip › supporting information/Fig 14(6).jpg]

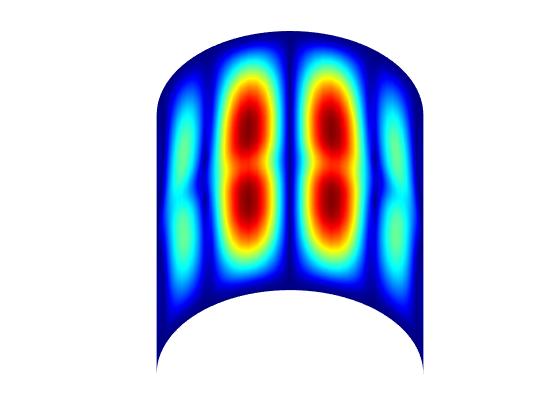

Supplement: S1 File — (ZIP) [file pone.0299586.s001.zip › supporting information/Fig 5(1).jpg]

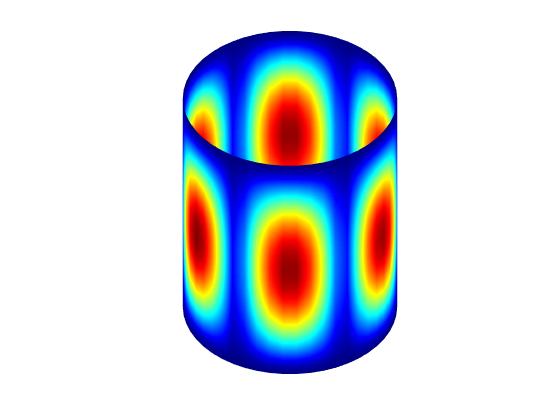

Supplement: S1 File — (ZIP) [file pone.0299586.s001.zip › supporting information/Fig 5(10).jpg]

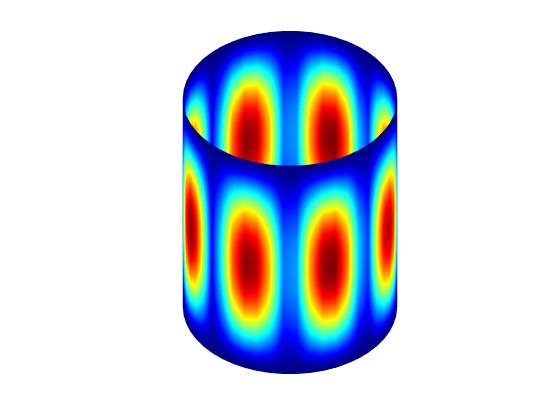

Supplement: S1 File — (ZIP) [file pone.0299586.s001.zip › supporting information/Fig 5(11).jpg]

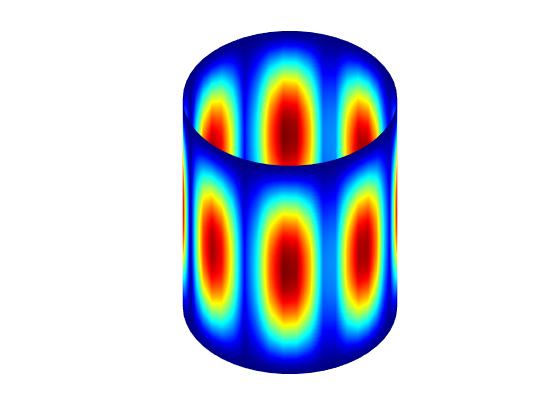

Supplement: S1 File — (ZIP) [file pone.0299586.s001.zip › supporting information/Fig 5(12).jpg]

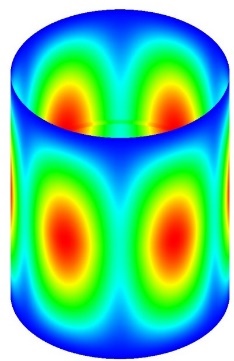

Supplement: S1 File — (ZIP) [file pone.0299586.s001.zip › supporting information/Fig 5(13).jpg]

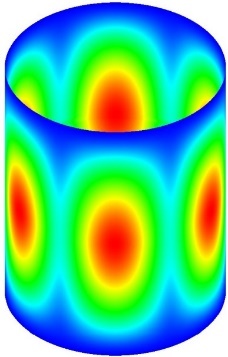

Supplement: S1 File — (ZIP) [file pone.0299586.s001.zip › supporting information/Fig 5(14).jpg]

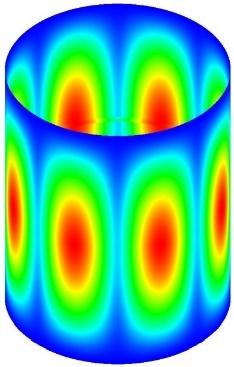

Supplement: S1 File — (ZIP) [file pone.0299586.s001.zip › supporting information/Fig 5(15).jpg]

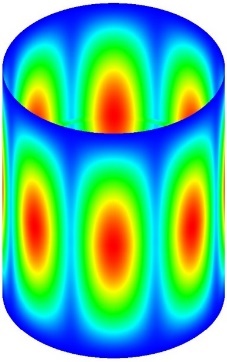

Supplement: S1 File — (ZIP) [file pone.0299586.s001.zip › supporting information/Fig 5(16).jpg]

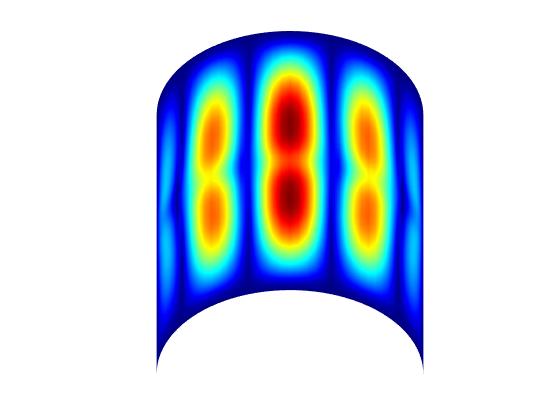

Supplement: S1 File — (ZIP) [file pone.0299586.s001.zip › supporting information/Fig 5(2).jpg]

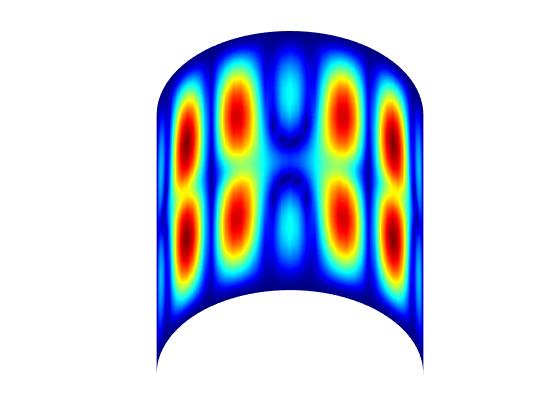

Supplement: S1 File — (ZIP) [file pone.0299586.s001.zip › supporting information/Fig 5(3).jpg]

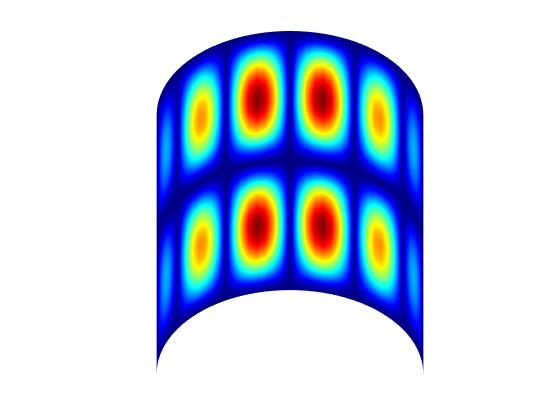

Supplement: S1 File — (ZIP) [file pone.0299586.s001.zip › supporting information/Fig 5(4).jpg]

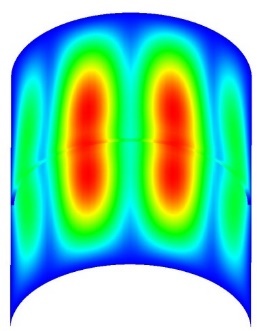

Supplement: S1 File — (ZIP) [file pone.0299586.s001.zip › supporting information/Fig 5(5).jpg]

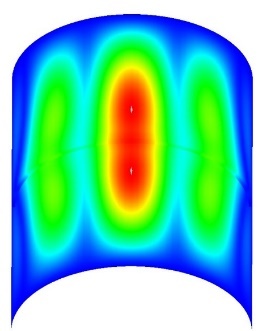

Supplement: S1 File — (ZIP) [file pone.0299586.s001.zip › supporting information/Fig 5(6).jpg]

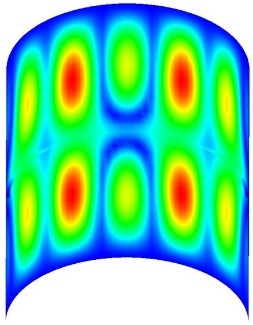

Supplement: S1 File — (ZIP) [file pone.0299586.s001.zip › supporting information/Fig 5(7).jpg]

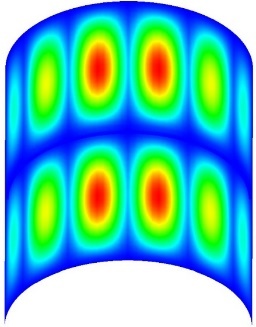

Supplement: S1 File — (ZIP) [file pone.0299586.s001.zip › supporting information/Fig 5(8).jpg]

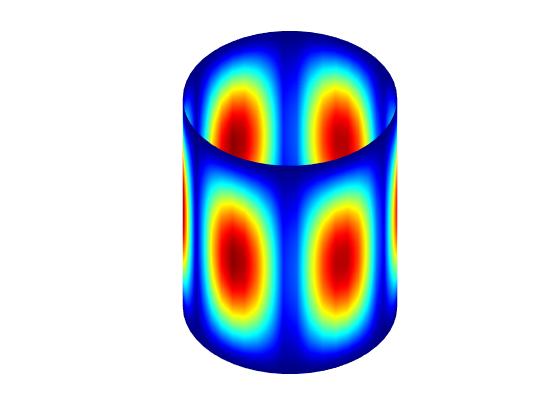

Supplement: S1 File — (ZIP) [file pone.0299586.s001.zip › supporting information/Fig 5(9).jpg]
