# Supplementary material for: Correction: The modeling method for vibration characteristics analysis of composite laminated rotationally stiffened shell
Source: PLoS One. 2024 Nov 13;19(11):e0313986. doi: 10.1371/journal.pone.0313986 (PMC11560018; doi:10.1371/journal.pone.0313986)
Supplement: S1 File — (PDF) [file pone.0313986.s001.pdf]

RESEARCH ARTICLE

The modeling method for vibration characteristics analysis of composite laminated rotationally stiffened shell

Dong Shi[1], **hong zhang**[2,3]*, **Yiqun Ding**[2], **Changrun Yang**[2], **Tianlin Cheng**[2]

1 VMTT INDUSTRY CO., LTD, Chongqing, PR China, **2** College of Mechanical and Electrical Engineering, Nanjing University of Aeronautics and Astronautics, Nanjing, PR China, **3** Jiangsu Key Laboratory of Precision and Micro-Manufacturing Technology, Nanjing University of Aeronautics and Astronautics, Nanjing, PR China

* zhanghongyuxin@outlook.com

Check for updates

OPEN ACCESS

Citation: Shi D, zhang hong, Ding Y, Yang C, Cheng T (2024) The modeling method for vibration characteristics analysis of composite laminated rotationally stiffened shell. PLoS ONE 19(6): e0299586. https://doi.org/10.1371/journal.pone.0299586

Editor: Waqas Saleem, TU Dublin Blanchardstown Campus: Technological University Dublin - Blanchardstown Campus, IRELAND

Received: December 9, 2023

Accepted: February 13, 2024

Published: June 18, 2024

Copyright: © 2024 Shi et al. This is an open access article distributed under the terms of the Creative Commons Attribution License, which permits unrestricted use, distribution, and reproduction in any medium, provided the original author and source are credited.

Data Availability Statement: All relevant data are within the paper and its Supporting Information files.

Funding: The authors gratefully acknowledge the financial support from the National Natural Science Foundation of China (Grant No. 52005255), Natural Science Foundation of Jiangsu Province of China (Grant No. BK20200430). Funders have provided financial assistance in the experimental portion of our paper. The funders had no role in study design,

Abstract

The composite laminated rotationally stiffened shell is widely applied in aviation, aerospace, ship, machinery and other fields. To investigate the vibration characteristics of composite laminated rotationally stiffened shells with varying elastic boundary conditions, a modeling method of composite laminated rotationally stiffened shells is established. Firstly, the first-order shear deformation theory (FSDT) and the modified Fourier series method are effectively applied to establish the allowable displacement function of the composite laminated rotationally stiffened shell. Secondly, the energy function of composite laminated rotationally stiffened shell is established, and the simulation of complex elastic boundary and coupling boundary is realized by using artificial virtual spring technology. Thirdly, the Rayleigh-Ritz method is used to solve the energy function. Finally, the vibration characteristics of composite laminated rotationally stiffened shells are obtained and analyzed. In the analysis of numerical results, the fast and uniform convergence of analysis modeling and the accuracy of the calculated results are verified. On this basis, the effect of some important parameters such as thickness-to-radius ratio and length-to-radius ratio of shell, boundary spring stiffness values, cone apex angle, thickness and width of laminated beams, number of stiffeners on the vibration characteristics of composite laminated rotationally stiffened shell is studied. In theory, it makes up for the vibration characteristics analysis of composite laminated rotationally stiffened shells. In practical application, it guides the noise reduction design of related structures.

1. Introduction

The composite laminated shell is basic structural elements in aviation, aerospace, ship and machinery, and other fields, which make it widely used in vehicle bodies, ship hull, and building houses. Reinforcing stiffeners are installed at appropriate positions of the shell through mechanical connections or adhesive bonding to effectively improve the overall strength and

data collection and analysis, decision to publish, or preparation of the manuscript.

Competing interests: The authors have declared that no competing interests exist.

rigidity of the structure. The addition of stiffeners leads to discontinuous changes in material, mass, and damping parameters at the interface between the shell and stiffeners. This results in complex variations in the waveform transformation and energy loss of vibration waves at the interface between the shell and stiffeners. Therefore, theoretical modeling and investigation of the vibration characteristics of composite laminated rotationally stiffened shells are of significant importance.

The early research on the vibration characteristics of rotationally stiffened shell structures primarily focused on conventional materials. Thein [1, 2] was one of the early scholars who studied the free vibration characteristics of rotationally stiffened plates and shells. Using finite difference algorithms, he derived the natural frequency equations for a circular shell reinforced with equidistant rings under tension. Weingarten [3] employed Galerkin's method and linear shell theory to predict and analyze the natural frequencies of simply supported conical shells reinforced with rings, validating the results through experimental studies. Najafi et al. [4] utilized the finite element method with axisymmetric elements to investigate the natural frequencies and mode shapes of thin cylindrical shells with circumferential stiffeners, treating each circumferential stiffener as a discrete element. Stanley et al. [5] employed a semi-analytical finite element method to study the natural frequencies of stiffened circular shells with simply supported boundary conditions, analyzing the influence of both longitudinal and circumferential stiffener quantities on the shell's natural frequencies. Sharma and Johns [6], based on Flügge shell theory and the Rayleigh-Ritz method, theoretically analyzed the free vibrations of rotationally stiffened circular shells with and without fixed boundary conditions.

Qu et al. [7] proposed an improved variational approach that applies all essential continuity constraints to the line interface using a modified variational principle and the least squares weighted residual method. Simultaneously, they established theoretical models for rotationally stiffened conical-cylindrical shells under different boundary conditions based on the Reissner-Naghdi thin shell theory combined with the discrete element stiffener theory. Chen et al. [8] introduced the concept of treating stiffeners with rectangular cross-sections as beams. They developed an analytical method for analyzing the free and forced vibration characteristics of rotationally stiffened conical shells with arbitrary boundary conditions.

Due to the widespread use of composite materials in various fields, research on the vibration characteristics of composite stiffened plate-shell structures has gradually advanced. Based on the kinematic nonlinearity of the high-order shear deformation theory and improved perturbation techniques, Shen et al. [9] investigated the substantial vibration behavior of nanocomposite stiffened cylindrical shells in a thermal environment. Li et al. [10], focusing on composite stiffened cylindrical shells, proposed a layered/solid element method based on the laminated theory and finite element method. They used this method to establish an analytical model for complex stiffened shell structures, resembling the geometric form of aircraft semi-hard shell-type fuselage structures. Sahoo [11] employed the finite element method to analyze the free vibration of composite laminated stiffened shallow spherical shells with cutouts. Eight-node curved quadratic isoparametric elements were used for the shell, and three-node beam elements were used for the stiffener, with a study on the influence of dimensions, boundary conditions, and the relative position of the shell center. Thomas and Roy [12], based on the first-order shear deformation theory assumption, used eight-node shell elements considering transverse shear effects to model composite material shell structures and conducted vibration analysis of functionally graded carbon nanotube-stiffened composite shell structures. Rout et al. [13] employed finite element methods to study the free vibration of composite stiffened cylindrical shells. The study used eight-node quadratic isoparametric shell elements, incorporating transverse shear deformation, rotational inertia, and three-node beam elements for the stiffeners, along with an analysis of the influence of lamination on the free vibration

characteristics of stiffened laminated cylindrical shells. Guo et al. [14] analyzed the free vibration characteristics of composite step-stiffened cylindrical shells with arbitrary boundary conditions using the first-order shear deformation theory and spectral-Chebyshev technique. They further investigated the impact of material parameters and geometric parameters on the vibration characteristics of composite stiffened shells. Abedini and Kiani [15] conducted an analytical study on the free vibration of composite graphene-stiffened cylindrical shells using the first-order shear deformation theory and Donnell motion relations. Dong et al. [16], based on the first-order shear deformation theory and Donnell motion relations, employed a novel Generalized Differential Quadrature (GDQ) method, combining it with the finite element method, to investigate the free vibration of the solid ring-stiffened conical shells.

In summary, although international scholars have conducted extensive research on the vibration characteristics of composite stiffened rotationally shell structures, the focus has often been on specific structural forms. When dealing with other structural forms, researchers are required to address complex and redundant modeling work. Therefore, establishing a unified analytical model for the vibration characteristics of composite laminated rotationally stiffened shells with complex boundaries is of significant importance.

In this article, a unified analysis model for the vibration characteristics of the composite laminated rotationally stiffened shell is established through the first-order shear deformation theory (FSDT) and the modified Fourier series method [17–19], specifically: firstly, the displacement admissible function is constructed using the modified Fourier series. Secondly, based on the first-order shear deformation theory, the domain energy generalization function of the composite laminated rotationally stiffened shell is established. Then, the equation of vibration characteristics is obtained by the Rayleigh-Ritz method, and the vibration characteristics of the structure are obtained. The rapid and consistent convergence of the theoretical modeling method is verified through the analysis of arithmetic cases, and the correctness of the vibration characteristics analysis model of the composite laminated rotationally stiffened shell is verified through finite element simulation and experimental tests. On this basis, important parameters are extracted and parametrically studied to obtain the influence of length ratio, boundary spring stiffness value, number of stiffeners, thickness, and width of stiffeners, and other significant parameters on the vibration characteristics of composite laminated rotationally stiffened shell, which provides the theoretical basis for vibration and the noise reduction of such structures.

2. Establishment of unified analysis model

2.1 Model description

This article mainly studies the vibration characteristics of composite laminated stiffened conical shells and cylindrical shells. Based on the geometric correlation, conical shells, and cylindrical shells can be uniformly represented by hyperbolic shell elements, as shown in Fig 1. The coordinates of the radial, circumferential, and normal directions of the hyperbolic shell element are represented by α, β, and z, respectively. R_α and R_β are the average curvature radius of α and β directions on the middle surface. L_α and L_β are length dimensions of α and β directions. L_z represents the height dimension of the z-coordinate direction on the middle surface. U, V, and W are displacements in α, β, and z directions. The composite laminated stiffened conical shell and cylindrical shell can be seen as a coupled structure of a rotating composite laminated shell structure and laminated curved beams. Fig 2 shows the coordinate system of these two types of structures. The coordinate system of shells is (o- z, θ, x), the coordinate of the nth laminated curved beam is located in the coordinate system (o_n- z_n, θ_n, x_n). The radius, thickness, and length of the middle surface of the laminated cylindrical shell are represented

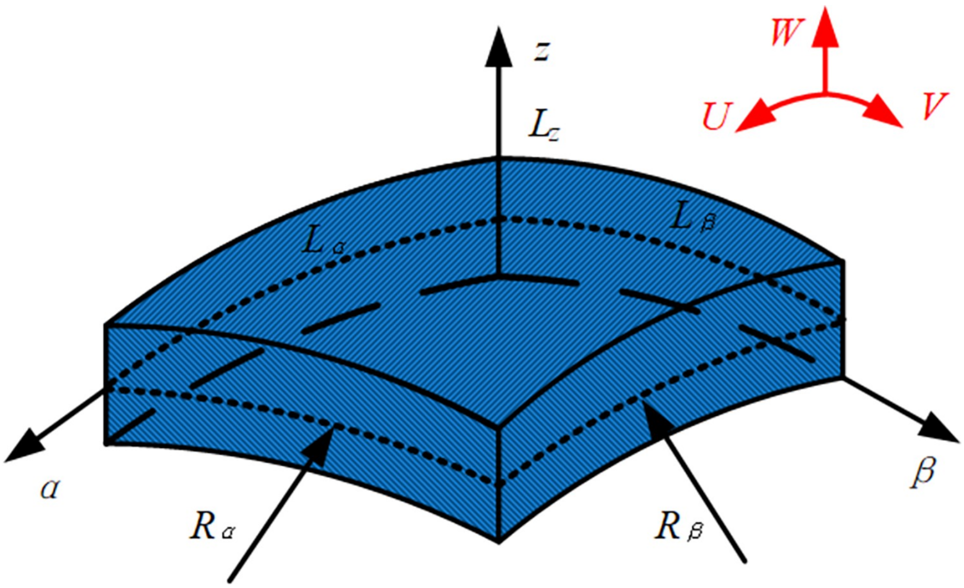

Fig 1. Geometric parameters and coordinate system of the cross-section of a hyperbolic shell element.

https://doi.org/10.1371/journal.pone.0299586.g001

by the symbols R_s, h_s, and L_s. The middle radius of the small end of the laminated conical shell is R_1, and the middle radius of the large end is R_2. The thickness, length, and cone top angle are h_s, $L_{s,}$ and φ. For laminated curved beam structures, the curvature radius, width, and thickness are represented by R_{bn}, $b_{n,}$ and h_n, respectively. Besides, the rotation angle of the entire

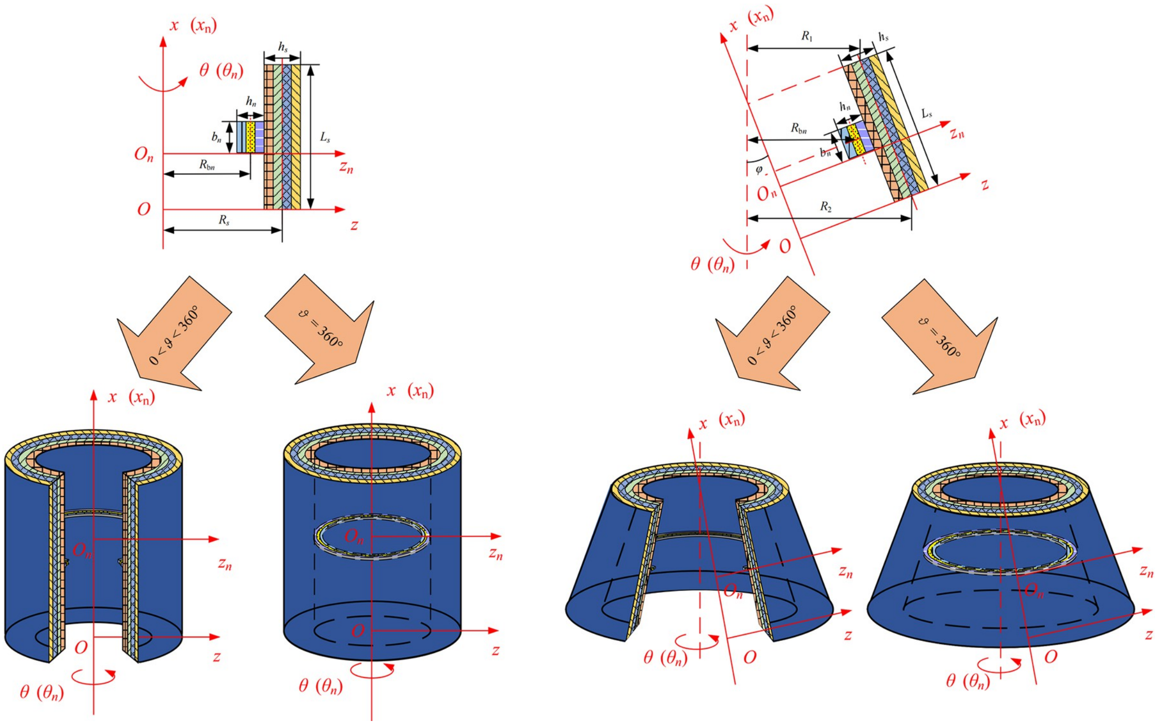

Fig 2. The coordinate and geometric description of unified model of composite laminated stiffened cylindrical and conical shells.

https://doi.org/10.1371/journal.pone.0299586.g002

Table 1. Parameters related to the conversion between cylindrical or conical shells.

The type of shell	Related parameters
Cylindrical shell	(1) $\alpha = x,\beta = \theta,A = 1,B = R_s,R_\alpha = \infty,R_\beta = R_s$<br>(2) $L_\alpha = L_s, L_\beta = \vartheta(0 \leq \vartheta \leq 360^\circ)$
Conical shell	(1) $\alpha = x,\beta = \theta,A = 1,B = x\sin\varphi,R_\alpha = \infty,R_\beta = x\tan\varphi$<br>(2) $L_\alpha = L_s, L_\beta = \vartheta(0 \leq \vartheta \leq 360^\circ)$

https://doi.org/10.1371/journal.pone.0299586.t001

stiffened shell is ϑ. When using hyperbolic shell structures to uniformly describe cylindrical or conical shells, the coordinate parameter transformation is given in Table 1.

The main purpose of this paper is to study the vibration characteristics of composite laminated stiffened cylindrical shells and conical shells with complex elastic boundary conditions. Therefore, artificial virtual spring technology has been cited to simulate boundary coupling between curved beams and shells, as well as the simulation of complex elastic boundary conditions. Next, Fig 3 takes the composite laminated stiffened conical shell as an example to show the spring combination settings for the support boundary and coupling boundary, respectively. As shown in Fig 3(A), to achieve simulation of elastic boundary conditions, three sets of continuously distributed linear springs (k_u, k_v, k_w) and two sets of torsional springs (K_θ, K_x) are introduced. It should be noted that when $\vartheta = 360^\circ$, the shell is defined as a closed shell. While in other cases, it is defined as an open shell. For closed stiffened shells, the stiffness of all boundary springs is set to zero at $\theta = 0^\circ$ and $\theta = 360^\circ$. Meanwhile, the coupling boundary springs are set at two edges of $\theta = 0^\circ$ and $\theta = 360^\circ$, as shown in Fig 3(B) and 3(C). For the

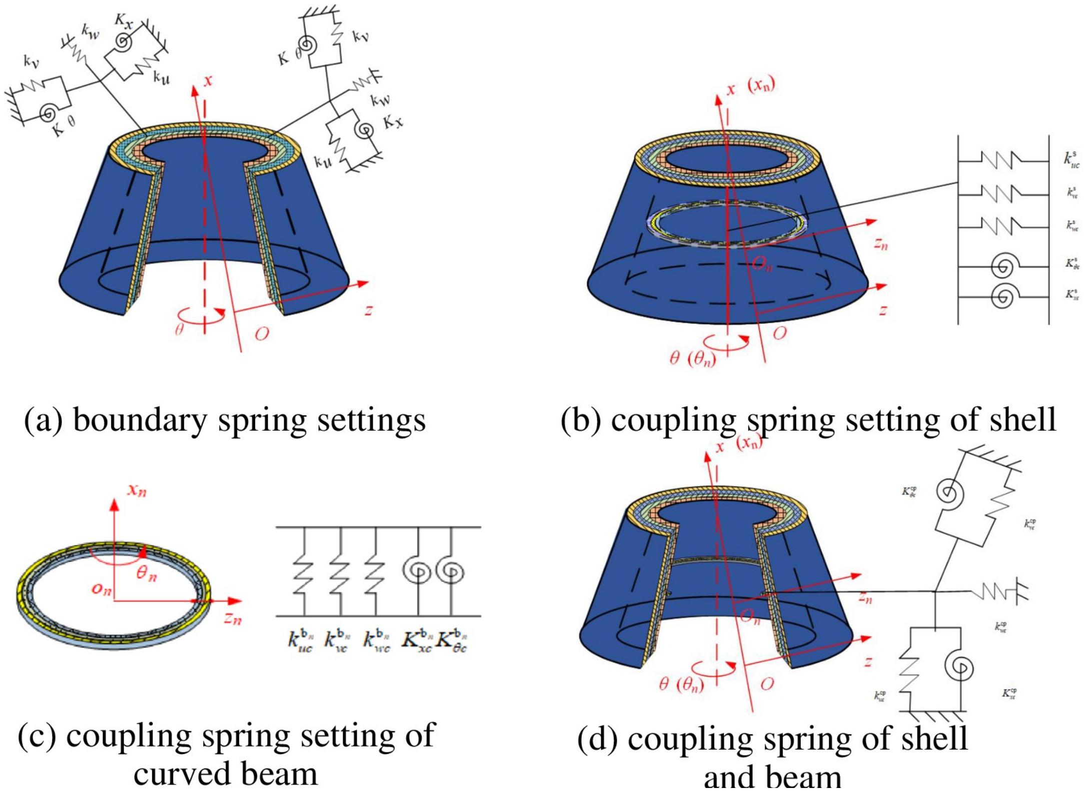

Fig 3. Boundary spring and coupling spring settings for composite laminated stiffened shell. (a) boundary spring settings. (b) coupling spring setting of shell. (c) coupling spring setting of curved beam. (d) coupling spring of shell and beam.

https://doi.org/10.1371/journal.pone.0299586.g003

closed shell, three sets of linear coupling springs (k_{uc}^{s}, k_{vc}^{s}, k_{wc}^{s}) and two sets of torsional springs ($K_{\theta c}^{\mathrm{s}}$, K_{xc}^{s}) are placed evenly on the coupling boundary. For the nth closed curved beams, three sets of linear coupling springs ($k_{uc}^{\mathrm{b}_n}$, $k_{vc}^{\mathrm{b}_n}$, $k_{wc}^{b_n}$) and two sets of torsional springs ($K_{\theta c}^{\mathrm{b}_n}$, $K_{xc}^{\mathrm{b}_n}$) also need to be placed at the coupling boundary. Fig 3(D) shows the uniformly arranged coupling springs between the rotating shell and the laminated curved beam, including three sets of linear coupling springs (k_{uc}^{cp}, k_{vc}^{cp}, k_{wc}^{cp}) and two sets of torsional springs ($K_{\theta c}^{\mathrm{cp}}$, K_{xc}^{cp}).

2.2 Construction of displacement admissible function and stress-strain relationship

According to the first order shear deformation theory (FSDT), the displacement components (U_{s}, V_{s}, W_{s}) at any point in the laminated shell and displacement components ($U_{\mathrm{b}n}$, $V_{\mathrm{b}n}$, $W_{\mathrm{b}n}$) at any point in laminated curved beam of the stiffened shell can be expressed as:

$$\begin{cases} U_{\mathrm{s}}(x,\theta,z,t) = u_{\mathrm{s}}(x,\theta,t) + z\phi_{x\mathrm{s}}(x,\theta,t) \\ V_{\mathrm{s}}(x,\theta,z,t) = v_{\mathrm{s}}(x,\theta,t) + z\phi_{\theta\mathrm{s}}(x,\theta,t) \\ W_{\mathrm{s}}(x,\theta,z,t) = w_{\mathrm{s}}(x,\theta,t) \end{cases} \tag{1}$$

$$\begin{cases} U_{\mathrm{b}n}(\theta_n,z,t) = u_{\mathrm{b}n}(\theta_n,t) + z\phi_{x\mathrm{b}n}(\theta_n,t) \\ V_{\mathrm{b}n}(\theta_n,z,t) = v_{\mathrm{b}n}(\theta_n,t) + z\phi_{\theta\mathrm{b}n}(\theta_n,t) \\ W_{\mathrm{b}n}(\theta_n,z,t) = w_{\mathrm{b}n}(\theta_n,t) \end{cases} \tag{2}$$

in which u_{s}, $v_{\mathrm{s},}$ and w_{s} represent the displacement along the x, θ and z directions on the reference surface of the laminated shell, respectively. Besides, $\phi_{x\mathrm{s}}$ and $\phi_{\theta\mathrm{s}}$ represent the transverse normal rotations of the reference surface with respect to the θ and x directions. $u_{\mathrm{b}n}$, $v_{\mathrm{b}n}$ and $w_{\mathrm{b}n}$ are the displacement along the θ_n, x_n and z_n directions on the reference surface of nth laminated curved beam, respectively. $\phi_{x\mathrm{b}n}$ and $\phi_{\theta\mathrm{b}n}$ donate the transverse normal rotations of the reference surface with respect to the θ and x directions. t is a time variable.

The improved Fourier series method can be used to establish the displacement admissible functions on the reference surface of composite laminated stiffened shell [20]. Their specific expressions are:

$$u_{\mathrm{s}}(x,\theta,t) = e^{-j\omega t}(\mathbf{\Phi}_u^{\mathrm{M}}(x,\theta) + \sum_{\mathrm{N}_q=1}^{2}\mathbf{\Phi}_u^{\mathrm{N}_q}(x,\theta))\mathbf{A}_{mn}$$

$$v_{\mathrm{s}}(x,\theta,t) = e^{-j\omega t}(\mathbf{\Phi}_v^{\mathrm{M}}(x,\theta) + \sum_{\mathrm{N}_q=1}^{2}\mathbf{\Phi}_v^{\mathrm{N}_q}(x,\theta))\mathbf{B}_{mn}$$

$$w_{\mathrm{s}}(x,\theta,t) = e^{-j\omega t}(\mathbf{\Phi}_w^{\mathrm{M}}(x,\theta) + \sum_{\mathrm{N}_q=1}^{2}\mathbf{\Phi}_w^{\mathrm{N}_q}(x,\theta))\mathbf{C}_{mn} \tag{3}$$

$$\phi_{x\mathrm{s}}(x,\theta,t) = e^{-j\omega t}(\mathbf{\Phi}_{\phi_r}^{\mathrm{M}}(x,\theta) + \sum_{\mathrm{N}_q=1}^{2}\mathbf{\Phi}_{\phi_x}^{\mathrm{N}_q}(x,\theta))\mathbf{D}_{mn}$$

$$\phi_{\theta\mathrm{s}}(x,\theta,t) = e^{-j\omega t}(\mathbf{\Phi}_{\phi_\theta}^{\mathrm{M}}(x,\theta) + \sum_{\mathrm{N}_q=1}^{2}\mathbf{\Phi}_{\phi_\theta}^{\mathrm{N}_q}(x,\theta))\mathbf{E}_{mn}$$

$$u_{bn}(\theta_n,t)=e^{-j\omega t}(\mathbf{\Psi}_{u_n}^{\Omega}(\theta_n)+\sum_{\Theta_q=1}^{2}\mathbf{\Psi}_{u_n}^{\Theta_q}(\theta_n))\mathbf{A}_l$$

$$v_{bn}(\theta_n,t)=e^{-j\omega t}(\mathbf{\Psi}_{v_n}^{\Omega}(\theta_n)+\sum_{\Theta_q=1}^{2}\mathbf{\Psi}_{v_n}^{\Theta_q}(\theta_n))\mathbf{B}_l$$

$$w_{bn}(\theta_n,t)=e^{-j\omega t}(\mathbf{\Psi}_{w_n}^{\Omega}(\theta_n)+\sum_{\Theta_q=1}^{2}\mathbf{\Psi}_{w_n}^{\Theta_q}(\theta_n))\mathbf{C}_l \quad (4)$$

$$\phi_{xbn}(\theta_n,t)=e^{-j\omega t}(\mathbf{\Psi}_{\phi_{xn}}^{\Omega}(\theta_n)+\sum_{\Theta_q=1}^{2}\mathbf{\Psi}_{\phi_{xn}}^{\Theta_q}(\theta_n))\mathbf{D}_l$$

$$\phi_{\theta bn}(\theta_n,t)=e^{-j\omega t}(\mathbf{\Psi}_{\phi_{\theta n}}^{\Omega}(\theta_n)+\sum_{\Theta_q=1}^{2}\mathbf{\Psi}_{\phi_{\theta n}}^{\Theta_q}(\theta_n))\mathbf{E}_l$$

where the displacement supplementary polynomial of composite laminated shells can be expressed as Φ^{M} and Φ^{Nq} ($N_q = 1, 2$). Besides, $\mathbf{A}_{mn}$, $\mathbf{B}_{mn}$, $\mathbf{C}_{mn}$, $\mathbf{D}_{mn}$, and $\mathbf{E}_{mn}$ represent unknown two-dimensional Fourier coefficient vectors for the displacement admissible function of laminated shells. The displacement supplementary polynomial of the nth laminated curved beam can be expressed as ${\Psi_n}^{\Omega}$ and ${\Psi_n}^{\Theta q}$ ($\Theta_q = 1, 2$). $\mathbf{A}_l$, $\mathbf{B}_l$, $\mathbf{C}_l$, $\mathbf{D}_l$ and $\mathbf{E}_l$ are unknown one-dimensional Fourier coefficient vectors for the displacement admissible function of the nth laminated curved beam. These parameters can be represented as:

$$\mathbf{\Phi}_u^{M}=\mathbf{\Phi}_w^{M}=\mathbf{\Phi}_v^{M}=\mathbf{\Phi}_{\phi_x}^{M}=\mathbf{\Phi}_{\phi_\theta}^{M}=\left\{\begin{array}{l}\cos\lambda_0^{\alpha}x\cos\lambda_0^{\beta}\theta,\cdots,\cos\lambda_0^{\alpha}x\cos\lambda_n^{\beta}\theta,\cdots,\\ \cos\lambda_0^{\alpha}x\cos\lambda_N^{b}\theta,\cdots,\cos\lambda_M^{\alpha}x\cos\lambda_N^{\beta}\theta\end{array}\right\}$$

$$\mathbf{\Phi}_u^{N_1}=\mathbf{\Phi}_v^{N_1}=\mathbf{\Phi}_w^{N_1}=\mathbf{\Phi}_{\phi_x}^{N_1}=\mathbf{\Phi}_{\phi_\theta}^{N_1}=\left\{\begin{array}{l}\sin(\lambda_{-2}^{\alpha}x)cos(\lambda_0^{\beta}\theta),\cdots,\sin(\lambda_{-2}^{\alpha}x)cos(\lambda_n^{\beta}\theta),\cdots,\\ \sin(\lambda_{-2}^{\alpha}x)cos(\lambda_N^{\beta}\theta),\cdots,\sin(\lambda_{-1}^{\alpha}x)cos(\lambda_N^{\beta}\theta)\end{array}\right\} \quad (5)$$

$$\mathbf{\Phi}_u^{N_2}=\mathbf{\Phi}_v^{N_2}=\mathbf{\Phi}_w^{N_2}=\mathbf{\Phi}_{\phi_x}^{N_2}=\mathbf{\Phi}_{\phi_\theta}^{N_2}=\left\{\begin{array}{l}cos(\lambda_0^{\alpha}x)\sin(\lambda_{-2}^{\beta}\theta),cos(\lambda_0^{\alpha}x)\sin(\lambda_{-1}^{\beta}\theta),\cdots,\\ cos(\lambda_m^{\alpha}x)\sin(\lambda_{-2}^{\beta}\theta),\cdots,cos(\lambda_M^{\alpha}x)\sin(\lambda_{-1}^{\beta}\theta)\end{array}\right\}$$

$$\mathbf{A}_{mn}=\left\{\begin{array}{l}A_{0,0}^{1},\cdots,A_{0,n}^{1},\cdots,A_{m,n}^{1},\cdots,A_{M,N}^{1},A_{-2,0}^{2},\cdots,A_{-2,n}^{2},\cdots,\\ A_{-2,N}^{2},\cdots,A_{-1,N}^{2},A_{0,-2}^{3},A_{0,-1}^{3},\cdots,A_{m,-2}^{3},\cdots,A_{M,-1}^{3}\end{array}\right\}^{T}$$

$$\mathbf{B}_{mn}=\left\{\begin{array}{l}B_{0,0}^{1},\cdots,B_{0,n}^{1},\cdots,B_{m,n}^{1},\cdots,B_{M,N}^{1},B_{-2,0}^{2},\cdots,B_{-2,n}^{2},\cdots,\\ B_{-2,N}^{2},\cdots,B_{-1,N}^{2},B_{0,-2}^{3},B_{0,-1}^{3},\cdots,B_{m,-2}^{3},\cdots,B_{M,-1}^{3}\end{array}\right\}^{T}$$

$$\mathbf{C}_{mn}=\left\{\begin{array}{l}C_{0,0}^{1},\cdots,C_{0,n}^{1},\cdots,C_{m,n}^{1},\cdots,C_{M,N}^{1},C_{-2,0}^{2},\cdots,C_{-2,n}^{2},\cdots,\\ C_{-2,N}^{2},\cdots,C_{-1,N}^{2},C_{0,-2}^{3},C_{0,-1}^{3},\cdots,C_{m,-2}^{3},\cdots,C_{M,-1}^{3}\end{array}\right\}^{T} \quad (6)$$

$$\mathbf{D}_{mn} = \left\{ \begin{array}{l} D^1_{0,0}, \cdots, D^1_{0,n}, \cdots, D^1_{m,n}, \cdots, D^1_{M,N}, D^2_{-2,0}, \cdots, D^2_{-2,n}, \cdots, \\ D^2_{-2,N}, \cdots, D^2_{-1,N}, D^3_{0,-2}, D^3_{0,-1}, \cdots, D^3_{m,-2}, \cdots, D^3_{M,-1} \end{array} \right\}^T$$

$$\mathbf{E}_{mn} = \left\{ \begin{array}{l} E^1_{0,0}, \cdots, E^1_{0,n}, \cdots, E^1_{m,n}, \cdots, E^1_{M,N}, E^2_{-2,0}, \cdots, E^2_{-2,n}, \cdots, \\ E^2_{-2,N}, \cdots, E^2_{-1,N}, E^3_{0,-2}, E^3_{0,-1}, \cdots, E^3_{m,-2}, \cdots, E^3_{M,-1} \end{array} \right\}^T$$

$$\boldsymbol{\Psi}^{\Omega}_{u_n} = \boldsymbol{\Psi}^{\Omega}_{v_n} = \boldsymbol{\Psi}^{\Omega}_{w_n} = \boldsymbol{\Psi}^{\Omega}_{\phi_{xn}} = \boldsymbol{\Psi}^{\Omega}_{\phi_{\theta n}} = \{\cos\lambda^{\alpha_n}_0 \theta_n, \cdots, \cos\lambda^{\alpha_n}_l \theta_n, \cdots \cos\lambda^{\alpha_n}_L \theta_n\}$$

$$\boldsymbol{\Psi}^{\Theta_1}_{u_n} = \boldsymbol{\Psi}^{\Theta_1}_{v_n} = \boldsymbol{\Psi}^{\Theta_1}_{w_n} = \boldsymbol{\Psi}^{\Theta_1}_{\phi_{xn}} = \boldsymbol{\Psi}^{\Theta_1}_{\phi_{\theta n}} = \frac{\alpha_n}{2\pi}\sin\left(\frac{\pi\theta_n}{2\alpha_n}\right) + \frac{\alpha_n}{2\pi}\sin\left(\frac{3\pi\theta_n}{2\alpha_n}\right) \tag{7}$$

$$\boldsymbol{\Psi}^{\Theta_2}_{u_n} = \boldsymbol{\Psi}^{\Theta_2}_{v_n} = \boldsymbol{\Psi}^{\Theta_2}_{w_n} = \boldsymbol{\Psi}^{\Theta_2}_{\phi_{xn}} = \boldsymbol{\Psi}^{\Theta_2}_{\phi_{\theta n}} = -\frac{\alpha_n}{2\pi}\cos\left(\frac{\pi\theta_n}{2\alpha_n}\right) + \frac{\alpha_n}{2\pi}\cos\left(\frac{3\pi\theta_n}{2\alpha_n}\right)$$

$$\mathbf{A}_l = \{A_0, A_1, \cdots, A_l, \cdots, A_L, a_1, a_2\}^T$$

$$\mathbf{B}_l = \{B_0, B_1, \cdots, B_l, \cdots, A_L, b_1, b_2\}^T$$

$$\mathbf{C}_l = \{C_0, C_1, \cdots, C_l, \cdots, C_L, c_1, c_2\}^T \tag{8}$$

$$\mathbf{D}_l = \{D_0, D_1, \cdots, D_l, \cdots, D_L, d_1, d_2\}^T$$

$$\mathbf{E}_l = \{E_0, E_1, \cdots, E_l, \cdots, E_L, e_1, e_2\}^T$$

where $\lambda^{\alpha}_m = m\pi/\alpha$, $\lambda^{\beta}_n = n\pi/\beta$ and $\lambda^{\alpha_n}_n = l\pi/\alpha_n$.

The normal strain and shear strain on composite laminated shells and curved beams are defined by the changes in strain and curvature of the reference surface:

$$\begin{array}{l} \boldsymbol{\varepsilon}^{s}_{\alpha} = \dfrac{1}{(1+z/R_{\alpha})}(\boldsymbol{\varepsilon}^{s0}_{\alpha} + z\chi^{s}_{\alpha});\ \gamma^{s}_{\alpha\beta} = \dfrac{1}{(1+z/R_{\alpha})}(\gamma^{s0}_{\alpha\beta} + z\chi^{s}_{\alpha\beta}) + \dfrac{1}{(1+z/R_{\beta})}(\gamma^{s0}_{\beta\alpha} + z\chi^{s}_{\beta\alpha}); \\ \boldsymbol{\varepsilon}^{s}_{\beta} = \dfrac{1}{(1+z/R_{\beta})}(\boldsymbol{\varepsilon}^{s0}_{\beta} + z\chi^{s}_{\beta});\ \gamma^{s}_{\alpha z} = \dfrac{1}{(1+z/R_{\alpha})}\gamma^{s0}_{\alpha z};\ \gamma^{s}_{\beta z} = \dfrac{1}{(1+z/R_{\beta})}\gamma^{s0}_{\beta z} \end{array} \tag{9}$$

$$\boldsymbol{\varepsilon}^{bn}_{\theta} = \boldsymbol{\varepsilon}^{bn0}_{\theta} + z\chi^{bn}_{\theta};\ \gamma^{bn}_{\theta x} = \gamma^{bn0}_{\theta x} + z\chi^{bn}_{\theta x};\ \gamma^{bn}_{\theta z} = \gamma^{bn0}_{\theta z};\ \gamma^{bn}_{xz} = \gamma^{bn0}_{xz} \tag{10}$$

in which $\boldsymbol{\varepsilon}^{s0}_{\alpha}$, $\boldsymbol{\varepsilon}^{s0}_{\beta}$, $\gamma^{s0}_{\alpha\beta}$, $\gamma^{s0}_{\beta\alpha}$, $\gamma^{s0}_{\alpha z}$ and $\gamma^{s0}_{\beta z}$ represent the strain component on the reference surface of laminated shells. χ^{s}_{α}, χ^{s}_{β}, $\chi^{s}_{\alpha\beta}$ and $\chi^{s}_{\beta\alpha}$ are curvature variation components on the reference surface of laminated shells. $\boldsymbol{\varepsilon}^{bn0}_{\theta}$, $\gamma^{bn0}_{\theta x}$, $\gamma^{bn0}_{\theta z}$ and γ^{bn0}_{xz} denote the strain component on the reference surface of the nth curved beam. χ^{bn}_{θ} and $\chi^{bn}_{\theta x}$ express curvature variation components on the

reference surface of the nth curved beam. The specific expressions for these components can be written as:

$$
\begin{aligned}
&\varepsilon_{\alpha}^{s0}=\frac{1}{A}\frac{\partial u_s}{\partial\alpha}+\frac{v_s}{AB}\frac{\partial A}{\partial\beta}+\frac{w_s}{R_\alpha};\quad \varepsilon_{\beta}^{s0}=\frac{1}{B}\frac{\partial v_s}{\partial\beta}+\frac{u_s}{AB}\frac{\partial B}{\partial\alpha}+\frac{w_s}{R_\beta};\quad \gamma_{\alpha\beta}^{s0}=\frac{1}{A}\frac{\partial v_s}{\partial\alpha}-\frac{u_s}{AB}\frac{\partial A}{\partial\beta};\\
&\gamma_{\beta\alpha}^{s0}=\frac{1}{B}\frac{\partial u_s}{\partial\beta}-\frac{v_s}{AB}\frac{\partial B}{\partial\alpha};\quad \gamma_{\alpha z}^{s0}=\frac{1}{A}\frac{\partial w_s}{\partial\alpha}-\frac{u_s}{R_\alpha}+\phi_{xs};\quad \gamma_{\beta z}^{s0}=\frac{1}{B}\frac{\partial w_s}{\partial\beta}-\frac{v_s}{R_\beta}+\phi_{\theta s};\\
&\chi_{\alpha}^{s}=\frac{1}{A}\frac{\partial\phi_{xs}}{\partial\alpha}+\frac{\phi_{\theta s}}{AB}\frac{\partial A}{\partial\beta};\quad \chi_{\beta}^{s}=\frac{1}{B}\frac{\partial\phi_{\theta s}}{\partial\beta}+\frac{\phi_{xs}}{AB}\frac{\partial B}{\partial\alpha};\quad \chi_{\alpha\beta}^{s}=\frac{1}{A}\frac{\partial\phi_{\theta s}}{\partial\alpha}-\frac{\phi_{xs}}{AB}\frac{\partial A}{\partial\beta};\\
&\chi_{\beta\alpha}^{s}=\frac{1}{B}\frac{\partial\phi_{xs}}{\partial\beta}-\frac{\phi_{\theta s}}{AB}\frac{\partial A}{\partial\alpha}
\end{aligned}
\tag{11}
$$

$$
\begin{aligned}
&\varepsilon_{\theta}^{bn0}=\frac{\partial v_{bn}}{R_n\partial\theta_n}+\frac{w_{bn}}{R_n};\quad \gamma_{\theta x}^{bn0}=\frac{\partial u_{bn}}{R_n\partial\theta_n};\quad \gamma_{xz}^{bn0}=\phi_{xbn};\\
&\gamma_{\theta z}^{p0}=\frac{\partial w_{bn}}{R_n\partial\theta_n}-\frac{v_{bn}}{R_n}+\phi_{\theta bn};\quad \chi_{\theta}^{bn}=\frac{\partial\phi_{\theta bn}}{R_n\partial\theta_n};\quad \chi_{\theta x}^{bn}=\frac{\partial\phi_{xbn}}{R_n\partial\theta_n}
\end{aligned}
\tag{12}
$$

The generalized forces of the laminated shell and nth curved beam in the stiffened shell are also obtained by integrating the strain, and their matrix form can be obtained as follows:

$$
\begin{bmatrix} N_\alpha^s\\ N_\beta^s\\ N_{\alpha\beta}^s\\ N_{\beta\alpha}^s\\ M_\alpha^s\\ M_\beta^s\\ M_{\alpha\beta}^s\\ M_{\beta\alpha}^s \end{bmatrix}
=
\begin{bmatrix}
A_{11} & A_{12} & A_{16} & A_{16} & B_{11} & B_{12} & B_{16} & B_{16}\\
A_{12} & A_{22} & A_{26} & A_{26} & B_{12} & B_{22} & B_{26} & B_{26}\\
A_{16} & A_{26} & A_{66} & A_{66} & B_{16} & B_{26} & B_{66} & B_{66}\\
A_{16} & A_{26} & A_{66} & A_{66} & B_{16} & B_{26} & B_{66} & B_{66}\\
B_{11} & B_{12} & B_{16} & B_{16} & D_{11} & D_{12} & D_{16} & D_{16}\\
B_{12} & B_{22} & B_{26} & B_{26} & D_{12} & D_{22} & D_{26} & D_{26}\\
B_{16} & B_{26} & B_{66} & B_{66} & D_{16} & D_{26} & D_{66} & D_{66}\\
B_{16} & B_{26} & B_{66} & B_{66} & D_{16} & D_{26} & D_{66} & D_{66}
\end{bmatrix}
\begin{bmatrix} \varepsilon_\alpha^{s0}\\ \varepsilon_\beta^{s0}\\ \gamma_{\alpha\beta}^{s0}\\ \gamma_{\beta\alpha}^{s0}\\ \chi_\alpha^s\\ \chi_\beta^s\\ \chi_{\alpha\beta}^s\\ \chi_{\beta\alpha}^s \end{bmatrix}
\tag{13}
$$

$$
\begin{bmatrix} Q_\beta^s\\ Q_\alpha^s \end{bmatrix} = \bar{\kappa}_s \begin{bmatrix} A_{44} & A_{45}\\ A_{45} & A_{55} \end{bmatrix} \begin{bmatrix} \gamma_{\beta z}^{s0}\\ \gamma_{\alpha z}^{s0} \end{bmatrix}
\tag{14}
$$

$$
\begin{bmatrix} N_\theta^{bn}\\ N_{\theta x}^{bn}\\ M_\theta^{bn}\\ M_{\theta x}^{bn} \end{bmatrix}
=
\begin{bmatrix}
A_{22} & A_{26} & B_{22} & B_{26}\\
A_{26} & A_{66} & B_{26} & B_{66}\\
B_{22} & B_{26} & D_{22} & D_{26}\\
B_{26} & B_{66} & D_{26} & D_{66}
\end{bmatrix}
\begin{bmatrix} \varepsilon_\theta^{bn0}\\ \gamma_{\theta x}^{bn0}\\ \chi_\theta^{bn}\\ \chi_{\theta x}^{bn} \end{bmatrix}
\tag{15}
$$

$$
\begin{bmatrix} Q_x^{bn}\\ Q_\theta^{bn} \end{bmatrix} = \bar{\kappa}_s \begin{bmatrix} A_{44} & A_{45}\\ A_{45} & A_{55} \end{bmatrix} \begin{bmatrix} \gamma_{xz}^{bn0}\\ \gamma_{\theta z}^{bn0} \end{bmatrix}
\tag{16}
$$

$$A_{ij} = \sum_{k=1}^{N_L} \bar{Q}_{ij}^k (Z_{k+1} - Z_k) \quad B_{ij} = \frac{1}{2}\sum_{k=1}^{N_L} \bar{Q}_{ij}^k (Z_{k+1}^2 - Z_k^2) \quad D_{ij} = \frac{1}{3}\sum_{k=1}^{N_L} \bar{Q}_{ij}^k (Z_{k+1}^3 - Z_k^3) \tag{17}$$

$$\begin{aligned}
\bar{Q}_{11}^{k} &= Q_{11}^k \cos^4\gamma^k + 2(Q_{12}^k + 2Q_{66}^k)\cos^2\gamma^k \sin^2\gamma^k + Q_{22}^k \sin^4\gamma^k \\
\bar{Q}_{12}^{k} &= (Q_{11}^k + Q_{22}^k - 4Q_{66}^k)\cos^2\gamma^k \sin^2\gamma^k + Q_{12}^k(\sin^4\gamma^k + \cos^4\gamma^k) \\
\bar{Q}_{22}^{k} &= Q_{11}^k \sin^4\gamma^k + 2(Q_{12}^k + 2Q_{66}^k)\cos^2\gamma^k \sin^2\gamma^k + Q_{22}^k \cos^4\gamma^k \\
\bar{Q}_{16}^{k} &= (Q_{11}^k - Q_{12}^k - 2Q_{66}^k)\cos^3\gamma^k \sin\gamma^k + (Q_{12}^k - Q_{22}^k + 2Q_{66}^k)\cos\gamma^k \sin^3\gamma^k \\
\bar{Q}_{26}^{k} &= (Q_{11}^k - Q_{12}^k - 2Q_{66}^k)\cos\gamma^k \sin^3\gamma^k + (Q_{12}^k - Q_{22}^k + 2Q_{66}^k)\cos^3\gamma^k \sin\gamma^k \\
\bar{Q}_{66}^{k} &= (Q_{11}^k + Q_{22}^k - 2Q_{12}^k - 2Q_{66}^k)\cos^2\gamma^k \sin^2\gamma^k + Q_{66}^k(\sin^4\gamma^k + \cos^4\gamma^k) \\
\bar{Q}_{44}^{k} &= Q_{44}^k \cos^2\gamma^k + Q_{55}^k \sin^2\gamma^k \\
\bar{Q}_{45}^{k} &= (Q_{55}^k - Q_{44}^k) cos\gamma^k \sin\gamma^k \\
\bar{Q}_{55}^{k} &= Q_{55}^k \cos^2\gamma^k + Q_{44}^k \sin^2\gamma^k
\end{aligned} \tag{18}$$

$$\begin{aligned}
Q_{11}^k &= \frac{E_1^k}{1-\mu_{12}^k\mu_{21}^k}, \quad Q_{12}^k = \mu_{21}^k Q_{11}^k, \quad Q_{22}^k = \frac{E_2^k}{1-\mu_{12}^k\mu_{21}^k} \\
Q_{44}^k &= G_{23}^k, \qquad Q_{55}^k = G_{13}^k, \qquad Q_{66}^k = G_{12}^k
\end{aligned} \tag{19}$$

where N_α^s, N_β^s, $N_{\alpha\beta}^s$ and $N_{\beta\alpha}^s$ express the normal and shear force resultants of the shell. M_α^s, M_β^s, $M_{\alpha\beta}^s$ and $M_{\beta\alpha}^s$ are the bending and twisting moment resultants of shell. Q_β^s and Q_α^s donate the transverse shear force resultants. For the *n*th laminated curved beam, the normal and shear force resultants are donated by N_θ^{bn} and $N_{\theta x}^{bn}$, and bending and twisting moment resultants are expressed by M_θ^{bn} and $M_{\theta x}^{bn}$. In addition, the transverse shear force resultants of the *n*th laminated curved beam are represented by the symbols Q_x^{bn} and Q_θ^{bn}. According to the FSDT, the shear correction coefficient $\bar{\kappa}_s$ is cited. N_L represents the number of layers of laminated shells or curved beams. For the *kth* layer, Z_{k+1} and Z_k are the coordinate values of the upper and lower surface thickness. In Eq (18), γ is the fiber angle of the *kth* layer. In Eq (19), the longitudinal Young's modulus and the transverse Young's modulus are expressed as E_1 and E_2, and the major Poisson's ratios are μ_{12} and μ_{21}, which can be determined by equation $\mu_{12}E_2 = \mu_{21}E_1$. In addition, G_{12}, G_{13}, and G_{23} are shear moduli, and by letting $E_1 = E_2$, $G_{12} = G_{13} = G_{23} = E_1/(2+2\mu_{12})$, it can be readily used to analyze isotropic material structure.

2.3 Energy equation and solution process

In the process of solving the vibration characteristics of composite laminated stiffened rotary shell with complex elastic boundary conditions, the establishment and solution process of the energy equation are obtained using the Rayleigh-Ritz method. Firstly, the specific expression for the Lagrangian equation is given:

$$L_S = T_S - U_S - U_{S-\text{coupling}} - U_{SP} - W_{S\&B_n} \tag{20}$$

$$L_{B_n} = T_{B_n} - U_{B_n} - U_{B_n-\text{coupling}} - W_{S\&B_n} \tag{21}$$

in which T_S and T_{B_n} (n = 1, 2,. . .) represent the total kinetic energy of the shell and *n*th curved

beam in a laminated stiffened shell. U_S and U_{B_n} are the total potential energy of the shell and nth curved beam. $U_{\text{S-coupling}}$ and $U_{\mathrm{B}_n-\text{coupling}}$ donate the coupling potential energy stored on the coupling boundary of the shell and nth curved beam when $\vartheta = 360^\circ$. In addition, U_{SP} is the spring potential energy stored on the boundaries of the laminated shell, and $W_{\mathrm{S\&B}_n}$ is the coupling potential energy when the laminated shell is coupled with the nth laminated curved beam. The specific expressions of these energy equations are given below.

The total kinetic energy T_{S} and T_{B_n} (n = 1, 2,. . .) can be written as follows:

$$T_{\mathrm{S}} = \frac{1}{2}\int_0^{R_{\mathrm{p}}}\int_0^{\vartheta}\left\{\begin{array}{l} I_{\mathrm{s}0}\left(\frac{\partial u_{\mathrm{s}}}{\partial t}\right)^2 + 2I_{\mathrm{s}1}\left(\frac{\partial u_{\mathrm{s}}}{\partial t}\right)\left(\frac{\partial \phi_{x\mathrm{s}}}{\partial t}\right) + I_{\mathrm{s}2}\left(\frac{\partial \phi_{x\mathrm{s}}}{\partial t}\right)^2 + I_{\mathrm{s}0}\left(\frac{\partial v_{\mathrm{s}}}{\partial t}\right)^2 \\ +2I_{\mathrm{s}1}\left(\frac{\partial v_{\mathrm{s}}}{\partial t}\right)\left(\frac{\partial \phi_{\theta\mathrm{s}}}{\partial t}\right) + I_{\mathrm{s}2}\left(\frac{\partial \phi_{\theta\mathrm{s}}}{\partial t}\right)^2 + I_{\mathrm{s}0}\left(\frac{\partial w_{\mathrm{s}}}{\partial t}\right)^2 \end{array}\right\} B dx d\theta \quad (22)$$

$$I_{\mathrm{s}0} = \sum_{k=1}^{N_L}\int_{Z_k}^{Z_{k+1}} \rho_{\mathrm{s}}^k dz; \quad I_{\mathrm{s}1} = \sum_{k=1}^{N_L}\int_{Z_k}^{Z_{k+1}} \rho_{\mathrm{s}}^k \cdot z dz; \quad I_{\mathrm{s}2} = \sum_{k=1}^{N_L}\int_{Z_k}^{Z_{k+1}} \rho_{\mathrm{s}}^k \cdot z^2 dz \quad (23)$$

$$T_{\mathrm{B}_n} = \frac{1}{2}\int_0^{\vartheta}\left\{\begin{array}{l} I_{\mathrm{b}n0}\left(\frac{\partial u_{\mathrm{b}n}}{\partial t}\right)^2 + 2I_{\mathrm{b}n1}\left(\frac{\partial u_{\mathrm{b}n}}{\partial t}\right)\left(\frac{\partial \phi_{x\mathrm{b}n}}{\partial t}\right) + I_{\mathrm{b}n2}\left(\frac{\partial \phi_{x\mathrm{b}n}}{\partial t}\right)^2 + I_{\mathrm{b}n0}\left(\frac{\partial v_{\mathrm{b}n}}{\partial t}\right)^2 \\ +2I_{\mathrm{b}n1}\left(\frac{\partial v_{\mathrm{b}n}}{\partial t}\right)\left(\frac{\partial \phi_{\theta\mathrm{b}n}}{\partial t}\right) + I_{\mathrm{b}n2}\left(\frac{\partial \phi_{\theta\mathrm{b}n}}{\partial t}\right)^2 + I_{\mathrm{b}n0}\left(\frac{\partial w_{\mathrm{b}n}}{\partial t}\right)^{22} \end{array}\right\} R_{\mathrm{b}n} d\theta_n \quad (24)$$

$$I_{\mathrm{b}n0} = \sum_{k=1}^{N_L}\int_{Z_k}^{Z_{k+1}} \rho_{\mathrm{b}n}^k dz_n; \quad I_{\mathrm{b}n1} = \sum_{k=1}^{N_L}\int_{Z_k}^{Z_{k+1}} \rho_{\mathrm{b}n}^k \cdot z_n dz_n; \quad I_{\mathrm{b}n2} = \sum_{k=1}^{N_L}\int_{Z_k}^{Z_{k+1}} \rho_{\mathrm{b}n}^k \cdot z_n^2 dz_n \quad (25)$$

where ρ_{s}^k is the material density of the *kth* layer for the laminated shell, and $\rho_{\mathrm{b}n}^k$ donates the material density of the *kth* layer for the laminated curved beam.

The specific expression of total kinetic energy U_{S} and U_{B_n} can be expressed represented as follows, where U_{S} includes the tensile potential energy U_{stretch}, the bending potential energy U_{bend}, and their coupling potential energy $U_{\text{s-b}}$.

$$U_{\mathrm{S}} = U_{\text{stretch}} + U_{\text{s-b}} + U_{\text{bend}} = \frac{1}{2}\int_0^{R_{\mathrm{p}}}\int_0^{\vartheta}\left\{\begin{array}{l} N_\alpha^{\mathrm{s}}\varepsilon_\alpha^{\mathrm{s}0} + N_\beta^{\mathrm{s}}\varepsilon_\beta^{\mathrm{s}0} + N_{\alpha\beta}^{\mathrm{s}}\gamma_{\alpha\beta}^{\mathrm{s}0} + N_{\beta\alpha}^{\mathrm{s}}\gamma_{\beta\alpha}^{\mathrm{s}0} + \\ M_\alpha^{\mathrm{s}}\chi_\alpha^{\mathrm{s}} + M_\beta^{\mathrm{s}}\chi_\beta^{\mathrm{s}} + M_{\alpha\beta}^{\mathrm{s}}\chi_{\alpha\beta}^{\mathrm{s}} + M_{\beta\alpha}^{\mathrm{s}}\chi_{\beta\alpha}^{\mathrm{s}} + \\ Q_\beta^{\mathrm{s}}\gamma_{\beta z}^{\mathrm{s}0} + Q_\alpha^{\mathrm{s}}\gamma_{\alpha z}^{\mathrm{s}0} \end{array}\right\} B dx d\theta \quad (26)$$

$$U_{\mathrm{stretch}}=\frac{1}{2}\int_0^{R_s}\int_0^{\vartheta}\left\{\begin{array}{l} A_{11}\left(\frac{1}{A}\frac{\partial u_s}{\partial\alpha}+\frac{v_s}{AB}\frac{\partial A}{\partial\beta}+\frac{w_s}{R_\alpha}\right)^2+A_{22}\left(\frac{1}{B}\frac{\partial v_s}{\partial\beta}+\frac{u_s}{AB}\frac{\partial B}{\partial\alpha}+\frac{w_s}{R_\beta}\right)^2 \\ +2A_{12}\left(\frac{1}{A}\frac{\partial u_s}{\partial\alpha}+\frac{v_s}{AB}\frac{\partial A}{\partial\beta}+\frac{w_s}{R_\alpha}\right)\left(\frac{1}{B}\frac{\partial v_s}{\partial\beta}+\frac{u_s}{AB}\frac{\partial B}{\partial\alpha}+\frac{w_s}{R_\beta}\right)+2A_{16}\left(\frac{1}{A}\frac{\partial v_s}{\partial\alpha}-\frac{u_s}{AB}\frac{\partial A}{\partial\beta}\right) \\ \times\left(\frac{1}{A}\frac{\partial u_s}{\partial\alpha}+\frac{v_s}{AB}\frac{\partial A}{\partial\beta}+\frac{w_s}{R_\alpha}\right)+2A_{16}\left(\frac{1}{A}\frac{\partial u_s}{\partial\alpha}+\frac{v_s}{AB}\frac{\partial A}{\partial\beta}+\frac{w_s}{R_\alpha}\right)\left(\frac{1}{B}\frac{\partial u_s}{\partial\beta}-\frac{v_s}{AB}\frac{\partial B}{\partial\alpha}\right) \\ +2A_{26}\left(\frac{1}{B}\frac{\partial v_s}{\partial\beta}+\frac{u_s}{AB}\frac{\partial B}{\partial\alpha}+\frac{w_s}{R_\beta}\right)\left(\frac{1}{A}\frac{\partial v_s}{\partial\alpha}-\frac{u_s}{AB}\frac{\partial A}{\partial\beta}\right)+2A_{26}\left(\frac{1}{B}\frac{\partial u_s}{\partial\beta}-\frac{v_s}{AB}\frac{\partial B}{\partial\alpha}\right) \\ \times\left(\frac{1}{B}\frac{\partial v_s}{\partial\beta}+\frac{u_s}{AB}\frac{\partial B}{\partial\alpha}+\frac{w_s}{R_\beta}\right)+A_{66}\left(\frac{1}{A}\frac{\partial v_s}{\partial\alpha}-\frac{u_s}{AB}\frac{\partial A}{\partial\beta}\right)^2+A_{66}\left(\frac{1}{B}\frac{\partial u_s}{\partial\beta}-\frac{v_s}{AB}\frac{\partial B}{\partial\alpha}\right)^2 \\ +\bar{\kappa}_s A_{44}\left(\frac{1}{B}\frac{\partial w_s}{\partial\beta}-\frac{v_s}{R_\beta}+\phi_{\theta s}\right)^2+\bar{\kappa}_s A_{55}\left(\frac{1}{A}\frac{\partial w_s}{\partial\alpha}-\frac{u_s}{R_\alpha}+\phi_{xs}\right)^2 \\ +2\bar{\kappa}_s A_{45}\left(\frac{1}{B}\frac{\partial w_s}{\partial\beta}-\frac{v_s}{R_\beta}+\phi_{\theta s}\right)\left(\frac{1}{A}\frac{\partial w_s}{\partial\alpha}-\frac{u_s}{R_\alpha}+\phi_{xs}\right) \end{array}\right\}Bdxd\theta \quad (27)$$

$$U_{\mathrm{s-b}}=\int_0^{R_s}\int_0^{\vartheta}\left\{\begin{array}{l} B_{11}\left(\frac{1}{A}\frac{\partial\phi_{xs}}{\partial\alpha}+\frac{\phi_{\theta s}}{AB}\frac{\partial A}{\partial\beta}\right)\left(\frac{1}{A}\frac{\partial u_s}{\partial\alpha}+\frac{v_s}{AB}\frac{\partial A}{\partial\beta}+\frac{w_s}{R_\alpha}\right)+B_{12}\left(\frac{1}{B}\frac{\partial\phi_{\theta s}}{\partial\beta}+\frac{\phi_{xs}}{AB}\frac{\partial B}{\partial\alpha}\right) \\ \times\left(\frac{1}{A}\frac{\partial u_s}{\partial\alpha}+\frac{v_s}{AB}\frac{\partial A}{\partial\beta}+\frac{w_s}{R_\alpha}\right)+B_{16}\left(\frac{1}{A}\frac{\partial\phi_{\theta s}}{\partial\alpha}-\frac{\phi_{xs}}{AB}\frac{\partial A}{\partial\beta}\right)\left(\frac{1}{A}\frac{\partial u_s}{\partial\alpha}+\frac{v_s}{AB}\frac{\partial A}{\partial\beta}+\frac{w_s}{R_\alpha}\right) \\ +B_{16}\left(\frac{1}{B}\frac{\partial\phi_{xs}}{\partial\beta}-\frac{\phi_{\theta s}}{AB}\frac{\partial A}{\partial\alpha}\right)\left(\frac{1}{A}\frac{\partial u_s}{\partial\alpha}+\frac{v_s}{AB}\frac{\partial A}{\partial\beta}+\frac{w_s}{R_\alpha}\right)+B_{12}\left(\frac{1}{A}\frac{\partial\phi_{xs}}{\partial\alpha}+\frac{\phi_{\theta s}}{AB}\frac{\partial A}{\partial\beta}\right) \\ \times\left(\frac{1}{B}\frac{\partial v_s}{\partial\beta}+\frac{u_s}{AB}\frac{\partial B}{\partial\alpha}+\frac{w_s}{R_\beta}\right)+B_{22}\left(\frac{1}{B}\frac{\partial\phi_{\theta s}}{\partial\beta}+\frac{\phi_{xs}}{AB}\frac{\partial B}{\partial\alpha}\right)\left(\frac{1}{B}\frac{\partial v_s}{\partial\beta}+\frac{u_s}{AB}\frac{\partial B}{\partial\alpha}+\frac{w_s}{R_\beta}\right) \\ +B_{26}\left(\frac{1}{A}\frac{\partial\phi_{\theta s}}{\partial\alpha}-\frac{\phi_{xs}}{AB}\frac{\partial A}{\partial\beta}\right)\left(\frac{1}{B}\frac{\partial v_s}{\partial\beta}+\frac{u_s}{AB}\frac{\partial B}{\partial\alpha}+\frac{w_s}{R_\beta}\right)+B_{26}\left(\frac{1}{B}\frac{\partial\phi_{xs}}{\partial\beta}-\frac{\phi_{\theta s}}{AB}\frac{\partial A}{\partial\alpha}\right) \\ \times\left(\frac{1}{B}\frac{\partial v_s}{\partial\beta}+\frac{u_s}{AB}\frac{\partial B}{\partial\alpha}+\frac{w_s}{R_\beta}\right)+B_{16}\left(\frac{1}{A}\frac{\partial\phi_{xs}}{\partial\alpha}+\frac{\phi_{\theta s}}{AB}\frac{\partial A}{\partial\beta}\right)\left(\frac{1}{A}\frac{\partial v_s}{\partial\alpha}-\frac{u_s}{AB}\frac{\partial A}{\partial\beta}\right) \\ +B_{26}\left(\frac{1}{B}\frac{\partial\phi_{\theta s}}{\partial\beta}+\frac{\phi_{xs}}{AB}\frac{\partial B}{\partial\alpha}\right)\left(\frac{1}{A}\frac{\partial v_s}{\partial\alpha}-\frac{u_s}{AB}\frac{\partial A}{\partial\beta}\right)+B_{66}\left(\frac{1}{A}\frac{\partial\phi_{\theta s}}{\partial\alpha}-\frac{\phi_{xs}}{AB}\frac{\partial A}{\partial\beta}\right) \\ \times\left(\frac{1}{A}\frac{\partial v_s}{\partial\alpha}-\frac{u_s}{AB}\frac{\partial A}{\partial\beta}\right)+B_{66}\left(\frac{1}{B}\frac{\partial\phi_{xs}}{\partial\beta}-\frac{\phi_{\theta s}}{AB}\frac{\partial A}{\partial\alpha}\right)\left(\frac{1}{A}\frac{\partial v_s}{\partial\alpha}-\frac{u_s}{AB}\frac{\partial A}{\partial\beta}\right) \\ +B_{16}\left(\frac{1}{A}\frac{\partial\phi_{xs}}{\partial\alpha}+\frac{\phi_{\theta s}}{AB}\frac{\partial A}{\partial\beta}\right)\left(\frac{1}{B}\frac{\partial u_s}{\partial\beta}-\frac{v_s}{AB}\frac{\partial B}{\partial\alpha}\right)+B_{26}\left(\frac{1}{B}\frac{\partial\phi_{\theta s}}{\partial\beta}+\frac{\phi_{xs}}{AB}\frac{\partial B}{\partial\alpha}\right) \\ \times\left(\frac{1}{B}\frac{\partial u_s}{\partial\beta}-\frac{v_s}{AB}\frac{\partial B}{\partial\alpha}\right)+B_{66}\left(\frac{1}{A}\frac{\partial\phi_{\theta s}}{\partial\alpha}-\frac{\phi_{xs}}{AB}\frac{\partial A}{\partial\beta}\right)\left(\frac{1}{B}\frac{\partial u_s}{\partial\beta}-\frac{v_s}{AB}\frac{\partial B}{\partial\alpha}\right) \\ +B_{66}\left(\frac{1}{B}\frac{\partial\phi_{xs}}{\partial\beta}-\frac{\phi_{\theta s}}{AB}\frac{\partial A}{\partial\alpha}\right)\left(\frac{1}{B}\frac{\partial u_s}{\partial\beta}-\frac{v_s}{AB}\frac{\partial B}{\partial\alpha}\right) \end{array}\right\}Bdrd\theta \quad (28)$$

$$U_{\mathrm{bend}}=\frac{1}{2}\int_{0}^{R_s}\int_{0}^{\vartheta}\left\{\begin{array}{l}D_{11}\left(\frac{1}{A}\frac{\partial\phi_{xs}}{\partial\alpha}+\frac{\phi_{\theta s}}{AB}\frac{\partial A}{\partial\beta}\right)^{2}+2D_{12}\left(\frac{1}{B}\frac{\partial\phi_{\theta s}}{\partial\beta}+\frac{\phi_{xs}}{AB}\frac{\partial B}{\partial\alpha}\right)\\ \times\left(\frac{1}{A}\frac{\partial\phi_{xs}}{\partial\alpha}+\frac{\phi_{\theta s}}{AB}\frac{\partial A}{\partial\beta}\right)+2D_{16}\left(\frac{1}{A}\frac{\partial\phi_{\theta s}}{\partial\alpha}-\frac{\phi_{xs}}{AB}\frac{\partial A}{\partial\beta}\right)\left(\frac{1}{A}\frac{\partial\phi_{xs}}{\partial\alpha}+\frac{\phi_{\theta s}}{AB}\frac{\partial A}{\partial\beta}\right)\\ +2D_{16}\left(\frac{1}{B}\frac{\partial\phi_{xs}}{\partial\beta}-\frac{\phi_{\theta s}}{AB}\frac{\partial A}{\partial\alpha}\right)\left(\frac{1}{A}\frac{\partial\phi_{xs}}{\partial\alpha}+\frac{\phi_{\theta s}}{AB}\frac{\partial A}{\partial\beta}\right)+D_{22}\left(\frac{1}{B}\frac{\partial\phi_{\theta s}}{\partial\beta}+\frac{\phi_{xs}}{AB}\frac{\partial B}{\partial\alpha}\right)^{2}\\ +2D_{26}\left(\frac{1}{A}\frac{\partial\phi_{\theta s}}{\partial\alpha}-\frac{\phi_{xs}}{AB}\frac{\partial A}{\partial\beta}\right)\left(\frac{1}{B}\frac{\partial\phi_{\theta s}}{\partial\beta}+\frac{\phi_{xs}}{AB}\frac{\partial B}{\partial\alpha}\right)+2D_{26}\left(\frac{1}{B}\frac{\partial\phi_{xs}}{\partial\beta}-\frac{\phi_{\theta s}}{AB}\frac{\partial A}{\partial\alpha}\right)\\ \times\left(\frac{1}{B}\frac{\partial\phi_{\theta s}}{\partial\beta}+\frac{\phi_{xs}}{AB}\frac{\partial B}{\partial\alpha}\right)+D_{66}\left(\frac{1}{A}\frac{\partial\phi_{\theta s}}{\partial\alpha}-\frac{\phi_{xs}}{AB}\frac{\partial A}{\partial\beta}\right)^{2}+D_{66}\left(\frac{1}{B}\frac{\partial\phi_{xs}}{\partial\beta}-\frac{\phi_{\theta s}}{AB}\frac{\partial A}{\partial\alpha}\right)^{2}\\ +2D_{66}\left(\frac{1}{B}\frac{\partial\phi_{xs}}{\partial\beta}-\frac{\phi_{\theta s}}{AB}\frac{\partial A}{\partial\alpha}\right)\left(\frac{1}{A}\frac{\partial\phi_{\theta s}}{\partial\alpha}-\frac{\phi_{xs}}{AB}\frac{\partial A}{\partial\beta}\right)\end{array}\right\}Bdxd\theta \quad (29)$$

$$U_{Bn}=\frac{1}{2}\int_{0}^{\vartheta}\left\{\begin{array}{l}A_{22}\left(\frac{\partial v_{bn}}{R_{bn}\partial\theta}+\frac{w_{bn}}{R_{bn}}\right)^{2}+2A_{26}\left(\frac{\partial u_{bn}}{R_{bn}\partial\theta}\right)\left(\frac{\partial v_{bn}}{R_{bn}\partial\theta}+\frac{w_{bn}}{R_{bn}}\right)+A_{66}\left(\frac{\partial u_{bn}}{R_{bn}\partial\theta}\right)^{2}\\ +\bar{\kappa}_{s}A_{44}(\phi_{xbn})^{2}+2\bar{\kappa}_{s}A_{45}\left(\frac{\partial w_{bn}}{R_{bn}\partial\theta}-\frac{v_{bn}}{R_{bn}}+\phi_{\theta bn}\right)(\phi_{xbn})\\ +\bar{\kappa}_{s}A_{55}\left(\frac{\partial w_{bn}}{R_{bn}\partial\theta}-\frac{v_{bn}}{R_{bn}}+\phi_{\theta bn}\right)^{2}+2B_{22}\left(\frac{\partial\phi_{\theta bn}}{R_{bn}\partial\theta}\right)\left(\frac{\partial v_{bn}}{R_{bn}\partial\theta}+\frac{w_{bn}}{R_{bn}}\right)\\ +2B_{26}\left(\frac{\partial\phi_{xbn}}{R_{bn}\partial\theta}\right)\left(\frac{\partial v_{bn}}{R_{bn}\partial\theta}+\frac{w_{bn}}{R_{bn}}\right)+2B_{26}\left(\frac{\partial\phi_{\theta bn}}{R_{bn}\partial\theta}\right)\left(\frac{\partial u_{bn}}{R_{bn}\partial\theta}\right)\\ +2B_{66}\left(\frac{\partial\phi_{xbn}}{R_{bn}\partial\theta}\right)\left(\frac{\partial u_{bn}}{R_{bn}\partial\theta}\right)+D_{22}\left(\frac{\partial\phi_{\theta bn}}{R_{bn}\partial\theta}\right)^{2}+D_{66}\left(\frac{\partial\phi_{xbn}}{R_{bn}\partial\theta}\right)^{2}\\ +2D_{26}\left(\frac{\partial\phi_{xbn}}{R_{bn}\partial\theta}\right)\left(\frac{\partial\phi_{\theta bn}}{R_{bn}\partial\theta}\right)\end{array}\right\}R_{bn}d\theta_{n} \quad (30)$$

The spring potential energy U_{SP} stored on the boundaries of the laminated shell is given below:

$$U_{SP}=U_{SP}^{\theta}+U_{SP}^{x} \quad (31)$$

$$U_{SP}^{\theta}=\frac{1}{2}\int_{0}^{\vartheta}\int_{-h_s/2}^{h_s/2}\left\{\begin{array}{l}[k_{x0}^{u}u_{s}^{2}+k_{x0}^{v}v_{s}^{2}+k_{x0}^{w}w_{s}^{2}+K_{x0}^{x}\phi_{xs}^{2}+K_{x0}^{\theta}\phi_{\theta s}^{2}]_{x=0}\\ +[k_{xL_s}^{u}u_{s}^{2}+k_{xL_s}^{v}v_{s}^{2}+k_{xL_s}^{w}w_{s}^{2}+K_{xL_s}^{x}\phi_{xs}^{2}+K_{xL_s}^{\theta}\phi_{\theta s}^{2}]_{x=L_s}\end{array}\right\}Bdzd\theta \quad (32)$$

$$U_{SP}^{x}=\frac{1}{2}\int_{0}^{L_s}\int_{-h_s/2}^{h_s/2}\left\{\begin{array}{l}[k_{\theta0}^{u}u_{s}^{2}+k_{\theta0}^{v}v_{s}^{2}+k_{\theta0}^{w}w_{s}^{2}+K_{\theta0}^{x}\phi_{xs}^{2}+K_{\theta0}^{\theta}\phi_{\theta s}^{2}]_{\theta=0}\\ +[k_{\theta\vartheta}^{u}u_{s}^{2}+k_{\theta\vartheta}^{v}v_{s}^{2}+k_{\theta\vartheta}^{w}w_{s}^{2}+K_{\theta\vartheta}^{x}\phi_{xs}^{2}+K_{\theta\vartheta}^{\theta}\phi_{\theta s}^{2}]_{\theta=\vartheta}\end{array}\right\}dzdx \quad (33)$$

When $\vartheta = 360^\circ$, the coupling potential energy $U_{\text{S-coupling}}$ and $U_{\text{B}_n-\text{coupling}}$ stored on the coupling boundary of the shell and nth curved beam can be written as:

$$U_{\text{S}-\text{coupling}} = \frac{1}{2}\int_0^{L_s}\int_{-h_s/2}^{h_s/2}\left\{\begin{array}{l} k_{uc}^{s}(u_s|_{\theta=360^\circ} - u_s|_{\theta=0})^2 + k_{vc}^{s}(v_s|_{\theta=360^\circ} - v_s|_{\theta=0})^2 \\ +k_{wc}^{s}(w_s|_{\theta=360^\circ} - w_s|_{\theta=0})^2 + K_{xc}^{s}(\phi_{xs}|_{\theta=360^\circ} - \phi_{xs}|_{\theta=0})^2 \\ +K_{\theta c}^{s}(\phi_{\theta s}|_{\theta=360^\circ} - \phi_{\theta s}|_{\theta=0})^2 \end{array}\right\} dzdx \quad (34)$$

$$U_{\text{B}_n-\text{coupling}} = \frac{1}{2}\int_0^{\vartheta}\int_{-h_n/2}^{h_n/2}\left\{\begin{array}{l} k_{uc}^{\text{b}n}(u_{\text{b}n}|_{\theta_n=360^\circ} - u_{\text{b}n}|_{\theta_n=0})^2 + k_{vc}^{\text{b}n}(v_{\text{b}n}|_{\theta_n=360^\circ} - v_{\text{b}n}|_{\theta_n=0})^2 \\ +k_{wc}^{\text{b}n}(w_{\text{b}n}|_{\theta_n=360^\circ} - w_{\text{b}n}|_{\theta_n=0})^2 + K_{xc}^{\text{b}n}(\phi_{x\text{b}n}|_{\theta_n=360^\circ} - \phi_{x\text{b}n}|_{\theta_n=0})^2 \\ +K_{\theta c}^{\text{b}n}(\phi_{\theta\text{b}n}|_{\theta_n=360^\circ} - \phi_{\theta\text{b}n}|_{\theta_n=0})^2 \end{array}\right\} dz_n \quad (35)$$

When the laminated shell is coupled with the nth laminated curved beam, the coupling potential energy $W_{\text{S\&B}_n}$ is expressed as:

$$W_{\text{S\&B}_n} = \frac{1}{2}\int_0^{\vartheta}\left\{\begin{array}{l} k_{uc}^{\text{cp}}(u_s|_{x=L_s} - u_{\text{b}n})^2 + k_{vc}^{\text{cp}}(v_s|_{x=L_s} - v_{\text{b}n})^2 + k_{wc}^{\text{cp}}(w_s|_{x=L_s} - w_{\text{b}n})^2 \\ +K_{xc}^{\text{cp}}(\phi_{xs}|_{x=L_s} - \phi_{x\text{b}n})^2 + K_{yc}^{\text{cp}}(\phi_{\theta s}|_{x=L_s} - \phi_{\theta\text{b}n})^2 \end{array}\right\} d\theta \quad (36)$$

According to Rayleigh-Ritz energy technology, bring the above energy equation into Eqs (20)-(21), and calculate the partial derivative of the energy equation L_{S} and L_{B_n} to the unknown Fourier coefficient matrix to zero, which can be expressed as:

$$\frac{\partial L_{\text{S}}}{\partial \mathbf{P}_{mn}} = \frac{\partial T_{\text{S}}}{\partial \mathbf{P}_{mn}} - \frac{\partial U_{\text{S}}}{\partial \mathbf{P}_{mn}} - \frac{\partial U_{\text{S}-\text{coupling}}}{\partial \mathbf{P}_{mn}} - \frac{\partial U_{\text{SP}}}{\partial \mathbf{P}_{mn}} - \frac{\partial W_{\text{S\&B}_n}}{\partial \mathbf{P}_{mn}} = \mathbf{0} \quad (37)$$

$$\frac{\partial L_{\text{B}_n}}{\partial \mathbf{Q}_l} = \frac{\partial T_{\text{B}_n}}{\partial \mathbf{Q}_l} - \frac{\partial U_{\text{B}_n}}{\partial \mathbf{Q}_l} - \frac{\partial U_{\text{B}_n-\text{coupling}}}{\partial \mathbf{Q}_l} - \frac{\partial W_{\text{P\&B}_n}}{\partial \mathbf{Q}_l} = \mathbf{0} \quad (38)$$

$$\mathbf{P}_{mn} = [\,\mathbf{A}_{mn} \quad \mathbf{B}_{mn} \quad \mathbf{C}_{mn} \quad \mathbf{D}_{mn} \quad \mathbf{E}_{mn}\,]^T \quad (39)$$

$$\mathbf{Q}_l = [\,\mathbf{A}_l \quad \mathbf{B}_l \quad \mathbf{C}_l \quad \mathbf{D}_l \quad \mathbf{E}_l\,]^T \quad (40)$$

where $\mathbf{P}_{mn}$ represents the two-dimensional unknown Fourier coefficient matrix of laminated shells, and $\mathbf{Q}_l$ represents the one-dimensional unknown Fourier coefficient matrix of laminated curved beams. Rewrite Eqs (37)-(38) into matrix form, which can be expressed as:

$$(\mathbf{K}_{\text{S}} - \omega^2\mathbf{M}_{\text{S}})\mathbf{P}_{mn} + \omega^2\mathbf{C}_{\text{B}_n\&\text{S}}\mathbf{Q}_l = \mathbf{0} \quad (41)$$

$$(\mathbf{K}_{\text{B}_n} - \omega^2\mathbf{M}_{\text{B}_n})\mathbf{Q}_l + \omega^2\mathbf{C}_{\text{S\&B}_n}\mathbf{P}_{mn} = \mathbf{0} \quad (42)$$

in which the stiffness matrix and mass matrix of the laminated shell are donated by $\mathbf{K}_{\text{S}}$ and $\mathbf{M}_{\text{S}}$. The stiffness matrix and mass matrix of the nth laminated curved beam are donated by $\mathbf{K}_{\text{B}_n}$ and $\mathbf{M}_{\text{B}_n}$. Besides, the coupling matrix between the nth laminated curved beam and the laminated shell is represented by symbol $\mathbf{C}_{\text{B}_n\&\text{S}}$, and $\mathbf{C}_{\text{S\&B}_n} = \mathbf{C}_{\text{B}_n\&\text{S}}{}^T$. Eqs (41)-(42) need to be

converted into a system of linear equations for solution, thus a simpler solution can be obtained:

$$(\mathbf{R}-\omega^2\mathbf{S})\mathbf{G}=\mathbf{0} \tag{43}$$

$$\mathbf{R}=\begin{pmatrix}\mathbf{K}_S & \mathbf{C}_{\mathrm{B}_n\&\mathrm{S}}\\ 0 & \mathbf{K}_{\mathrm{B}_n}\end{pmatrix} \tag{44}$$

$$\mathbf{S}=\begin{pmatrix}\mathbf{M}_{\mathrm{S}} & 0\\ -\mathbf{C}_{\mathrm{S}\&\mathrm{B}_n} & \mathbf{M}_{\mathrm{B}_n}\end{pmatrix} \tag{45}$$

In this way, by solving the generalized eigenvalues, the natural frequencies of laminated stiffened shells can be obtained. Then the unknown Fourier coefficient vectors of the displacement functions can be gained by solving generalized eigenvectors. By substituting the unknown coefficient vectors into the expression of displacement admissible functions (1)-(2), the modal shapes of composite laminated stiffened shell can be obtained further.

3. Numerical results and discussions

This section conducts numerical discussions and results analysis based on the established rotational composite material stiffened shell model, to further obtain the vibration characteristics of composite laminated stiffened conical shells and cylindrical shells with complex elastic support boundaries. It mainly includes two parts: convergence and correctness verification analysis, as well as free vibration analysis. The composite materials used in this paper mainly include graphite-fiber-stiffened resin and glass-stiffened epoxy resin. The material parameters of graphite-fiber-stiffened resin are: E_1 = 1.85×10^{11}Pa, E_2 = 1.09×10^{10}Pa, G_{23} = 7.3×10^{9}Pa, G_{12} = 7.3×10^{9}Pa, G_{13} = 7.3×10^{9}Pa, μ_{12} = 0.28, ρ = 1600kg/m^3. The material parameters of glass-stiffened epoxy resin are: E_1 = 3.9×10^{10}Pa, E_2 = 8.3×10^{9}Pa, G_{23} = 4.1×10^{10}Pa, G_{12} = 4.1×10^{10}Pa, G_{13} = 4.1×10^{10}Pa, μ_{12} = 0.26, ρ = 1810kg/m^3 [21]. In addition, this model can also calculate the vibration characteristics of isotropic materials. Steel is used for calculation and analysis here, and its material parameters are: E_1 = E_2 = 2.16×10^{11}Pa, G_{23} = G_{12} = G_{13} = 8.31×10^{10}Pa, μ_{12} = 0.3, ρ = 7800kg/m^3. Without specific explanation, the dimensionless natural frequency parameter is defined as $\Omega=\omega/h_s\sqrt{\rho_s/E_1}$. The four boundary conditions of freedom, simple support, fixed support, and elasticity can be described by a combination of four symbols (F, S, C, and E). For example, FSCE is classified as free supported boundary at x = 0, simply supported boundary at x = L_s, fixed supported boundary at θ = 0, and elastic supported boundary at θ = ϑ.

3.1 Model validation

Perform the convergence analysis and spring stiffness research on the established composite laminated stiffened shell model. This facilitates the confirmation of the truncation values of the displacement functions for laminated shells and laminated curved beams in the stiffened shell, while also finding suitable boundary and coupling spring stiffness values in the stiffened shell model. Table 2 shows the first eight natural frequencies of stiffened cylindrical shells obtained by this method under different truncation values of laminated shells and curved beams. The truncation values of the shell are M_s and N_s. The truncation value of the nth curved beam is M_b. In this example, two types of shells are given: open stiffened cylindrical shell and closed stiffened cylindrical shell. Their identical geometric parameters are: R_s = 2m and L_s =

Table 2. Convergence analysis of the frequency parameter Ω of laminated stiffened cylindrical shells under fixed boundary conditions.

M_s×N_s	M_b	Mode number							
		1	2	3	4	5	6	7	8
		Open stiffened cylindrical shell: ϑ = 180˚, h_s = 0.02m, b_1 = 0.04m, h_1 = 0.06m, CCCC							
6×6	25	2.160	2.240	3.012	3.466	3.616	3.973	4.057	4.276
	50	2.160	2.240	3.012	3.466	3.615	3.973	4.057	4.275
10×10	25	2.014	2.019	2.425	2.460	2.466	2.491	2.870	2.896
	50	2.014	2.019	2.425	2.460	2.466	2.491	2.870	2.896
14×14	25	1.984	1.996	2.363	2.431	2.444	2.466	2.775	2.815
	50	1.984	1.996	2.363	2.431	2.444	2.466	2.775	2.815
18×18	25	1.979	1.986	2.343	2.418	2.427	2.466	2.759	2.798
	50	1.979	1.986	2.343	2.418	2.427	2.466	2.759	2.798
FEM		2.012	2.063	2.401	2.415	2.421	2.450	2.788	2.790
		closed stiffened cylindrical shell: ϑ = 360˚, h_s = 0.08m, b_1 = 0.06m, h_1 = 0.08m, CC							
6×6	25	0.628	0.629	0.707	0.709	1.016	1.026	1.086	1.112
	50	0.627	0.627	0.707	0.709	1.016	1.022	1.086	1.112
10×10	25	0.573	0.577	0.653	0.659	0.703	0.705	0.971	0.980
	50	0.573	0.577	0.653	0.659	0.703	0.705	0.971	0.980
14×14	25	0.571	0.572	0.642	0.646	0.702	0.702	0.872	0.879
	50	0.571	0.572	0.642	0.646	0.702	0.702	0.872	0.879
18×18	25	0.570	0.571	0.640	0.642	0.702	0.702	0.865	0.867
	50	0.570	0.571	0.640	0.642	0.702	0.702	0.865	0.867
FEM		0.557	0.557	0.649	0.649	0.678	0.678	0.901	0.901

https://doi.org/10.1371/journal.pone.0299586.t002

5m. The material of the laminated shell is graphite-fiber-stiffened resin, with a laying angle of [0˚/90˚] [23, 24]. In stiffened cylindrical shell, the number of curved beam is one, located at L_s/2, and the material used is steel. In addition, the frequencies obtained using the finite element method (FEM) are also given in this table for comparison with the frequencies obtained using the present method. From Table 2, it can be seen that when the truncation values are M_s×N_s = 18×18 and M_b = 25, the natural frequencies of the stiffened cylindrical shell can achieve convergence. At this point, the maximum error between the natural frequencies of each order obtained by the present method and the frequencies obtained by FEM is 3.77%. From this example, it can be seen that the present method has fast and uniform convergence, as well as accuracy.

Table 3 shows the first eight natural frequencies of composite laminated stiffened conical shells under different truncation values. In this example, the same geometric parameters as open conical shells and closed conical shells are: R_1 = 1m, L_s = 2m, φ = 15˚. The material of the laminated conical shell is graphite-fiber-stiffened resin, with a laying angle of [0˚/90˚]. In stiffened conical shell, the number of curved beam is one, located at L_s/2, and the material used is steel. From Table 3, it can be seen that the natural frequency of the stiffened conical shell can converge when M_s×N_s = 18×18 and M_b = 25. Therefore, in the subsequent example analysis, the truncation values are determined as M_s×N_s = 18×18 and M_b = 25.

From Tables 2 and 3, it is also found that the truncation value of the laminated curved beam has a relatively small impact on the convergence because there is only one laminated curved beam. Therefore, further analysis is needed to investigate the effect of the truncation value of the laminated shell on the convergence. Fig 4 shows the variation curves of the natural frequency parameter Ω of stiffened conical shells under different truncation values. It is not

Table 3. Convergence analysis of the frequency parameter Ω of laminated stiffened conical shells.

$M_s \times N_s$	M_b	Modal order							
		1	2	3	4	5	6	7	8
		Open stiffened conical shell: $\vartheta = 180^\circ$, $h_s = 0.02$m, $b_1 = 0.04$m, $h_1 = 0.06$m, CCCC							
6×6	25	7.091	9.449	9.745	10.294	10.723	11.721	13.952	15.595
	50	7.091	9.449	9.745	10.294	10.723	11.721	13.952	15.596
10×10	25	6.692	7.589	7.594	8.298	8.372	8.534	8.608	9.331
	50	6.692	7.589	7.594	8.298	8.372	8.534	8.608	9.332
14×14	25	6.564	7.482	7.503	8.004	8.110	8.367	8.430	8.856
	50	6.563	7.483	7.503	8.004	8.110	8.367	8.430	8.857
18×18	25	6.513	7.442	7.464	7.950	8.058	8.307	8.366	8.789
	50	6.513	7.442	7.464	7.950	8.058	8.307	8.366	8.789
		Closed stiffened conical shell: $\vartheta = 360^\circ$, $h_s = 0.08$m, $b_1 = 0.06$m, $h_1 = 0.08$m, SS							
6×6	25	1.531	2.648	2.648	3.575	3.580	3.600	3.792	4.012
	50	1.531	2.648	2.648	3.575	3.580	3.600	3.792	4.012
10×10	25	1.519	2.564	2.564	3.284	3.288	3.355	3.357	3.506
	50	1.519	2.564	2.564	3.284	3.288	3.355	3.357	3.506
14×14	25	1.513	2.535	2.535	3.257	3.257	3.337	3.338	3.379
	50	1.513	2.535	2.535	3.257	3.257	3.337	3.338	3.379
18×18	25	1.510	2.521	2.521	3.244	3.245	3.328	3.329	3.362
	50	1.510	2.521	2.521	3.244	3.245	3.328	3.329	3.362

https://doi.org/10.1371/journal.pone.0299586.t003

difficult to see that the natural frequency parameter Ω of open and closed stiffened shells tends to stabilize with the increase of truncation values.

Taking a stiffened cylindrical shell as an example, Fig 5 shows the mode shape diagrams of the stiffened shells obtained by the present method and the FEM. The geometric and material parameters of the stiffened cylindrical shells are consistent with Table 2. From Fig 5, it can be observed that the results of present method correspond to those of the FEM.

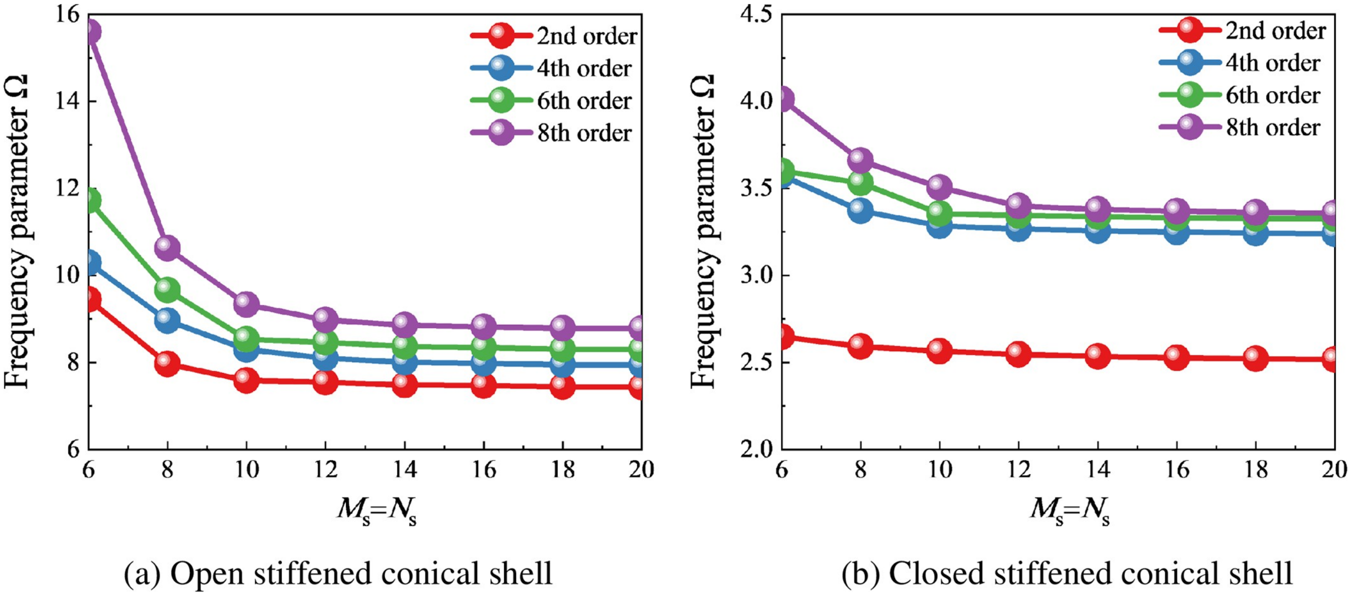

Fig 4. The variation curve of frequency parameter Ω for stiffened conical shells under different truncation values. (a) Open stiffened conical shell. (b) Closed stiffened conical shell.

https://doi.org/10.1371/journal.pone.0299586.g004

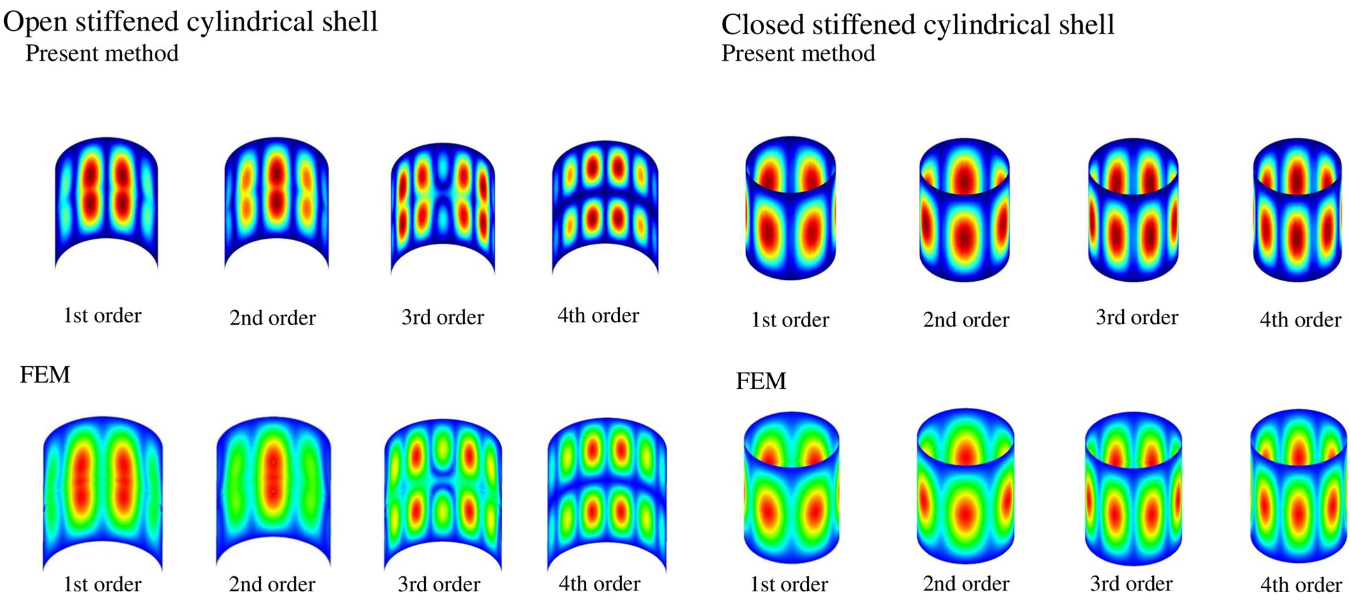

Fig 5. Mode shapes of the composite laminated stiffened cylindrical shell. Open stiffened cylindrical shell: Present method - 1st order, 2nd order, 3rd order, 4th order. FEM - 1st order, 2nd order, 3rd order, 4th order; Closed stiffened cylindrical shell: Present method - 1st order, 2nd order, 3rd order, 4th order. FEM - 1st order, 2nd order, 3rd order, 4th order.

https://doi.org/10.1371/journal.pone.0299586.g005

This study employs the artificial virtual spring technique to simulate arbitrary boundary conditions and rigid continuity conditions in coupling locations. Therefore, it is necessary to choose a reasonable spring stiffness value. Virtual spring groups, including linear springs $k(k_u, k_v, k_w)$, torsional springs $K(K_\theta, K_x)$, shell internal coupling springs k_c (k^s_{uc}, k^s_{vc}, k^s_{wc}, $K^s_{\theta c}$, K^s_{xc}), and coupling springs between the shell and beams k_{cp} (k^{cp}_{uc}, k^{cp}_{vc}, k^{cp}_{wc}, $K^{cp}_{\theta c}$, K^{cp}_{xc}) are arranged at the support or coupling boundaries. Fig 6 takes an open composite cylindrical shell as an example and provides the variation curves of the first four frequency parameters under different boundary spring stiffness values, with material parameters and geometric parameters consistent with those in case of Table 2.

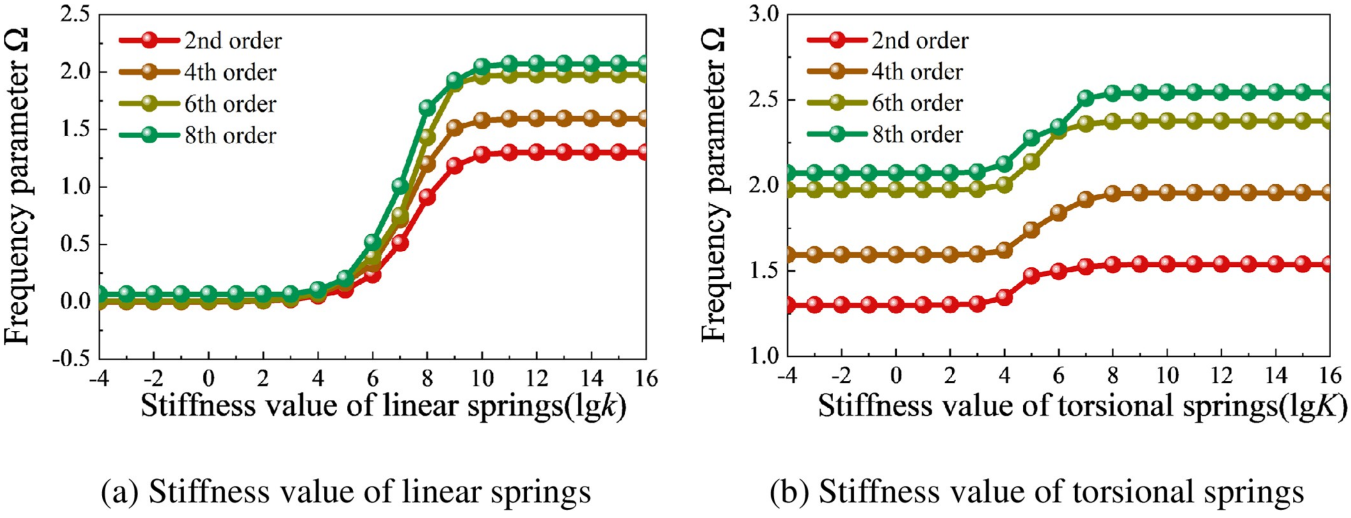

Fig 6. Variation curves of the first four frequency parameter Ω of the open composite cylindrical shell under different boundary spring stiffness values. (a) Stiffness value of linear springs. (b) Stiffness value of torsional springs.

https://doi.org/10.1371/journal.pone.0299586.g006

Table 4. The stiffness value of the boundary spring under different boundary conditions.

Boundary condition	Stiffness value of the boundary spring				
	k_u	k_v	k_w	K_r	K_θ
C	10^{16}	10^{16}	10^{16}	10^{16}	10^{16}
S	10^{16}	10^{16}	10^{16}	0	0
F	0	0	0	0	0
E^1	10^8	10^8	10^8	10^8	10^8
E^2	10^6	10^6	10^6	0	0

https://doi.org/10.1371/journal.pone.0299586.t004

Fig 6(A) shows the influence curve of linear spring stiffness variation on the frequency parameter Ω of the cylindrical shell when the torsional spring stiffness value K is zero. From the figure, it can be observed that when the linear boundary spring stiffness value k is in the range of 10^{-4} to 10^3, it can simulate free boundary (F). When the linear spring stiffness value k increases to 10^{11}, the numerical values of the frequency parameters begin to stabilize, simulating simply supported boundary (S). When the linear spring stiffness value k is in the range of 10^3 to 10^{10}, it achieves the simulation of elastic boundary conditions.

In Fig 6(B), the linear boundary spring stiffness value is kept at 10^{16}, and the frequency parameter Ω of the cylindrical shell increases with the increase of torsional spring stiffness value K. It stabilizes when K reaches 10^8, at which point the boundary conditions of the cylindrical shell can be considered as clamped boundary (C). Based on the content in Fig 6, the boundary spring stiffness values under different boundary conditions are shown in Table 4, which also includes the stiffness values of two elastic boundary conditions.

Taking a closed composite cylindrical shell as an example, Fig 7 illustrates the influence curves of frequency parameters with varying shell internal coupling spring stiffness values under boundary conditions CC (clamped-clamped) and SS (simply supported-simply supported). The material parameters and geometric parameters of the closed cylindrical shell are referenced from the values in Table 2. In Fig 7, it can be observed that when the shell internal coupling spring stiffness value k_c is less than 10^4, the influence of the shell internal coupling spring on the structural frequency parameters is small. When k_c is greater than 10^4, the frequency parameters

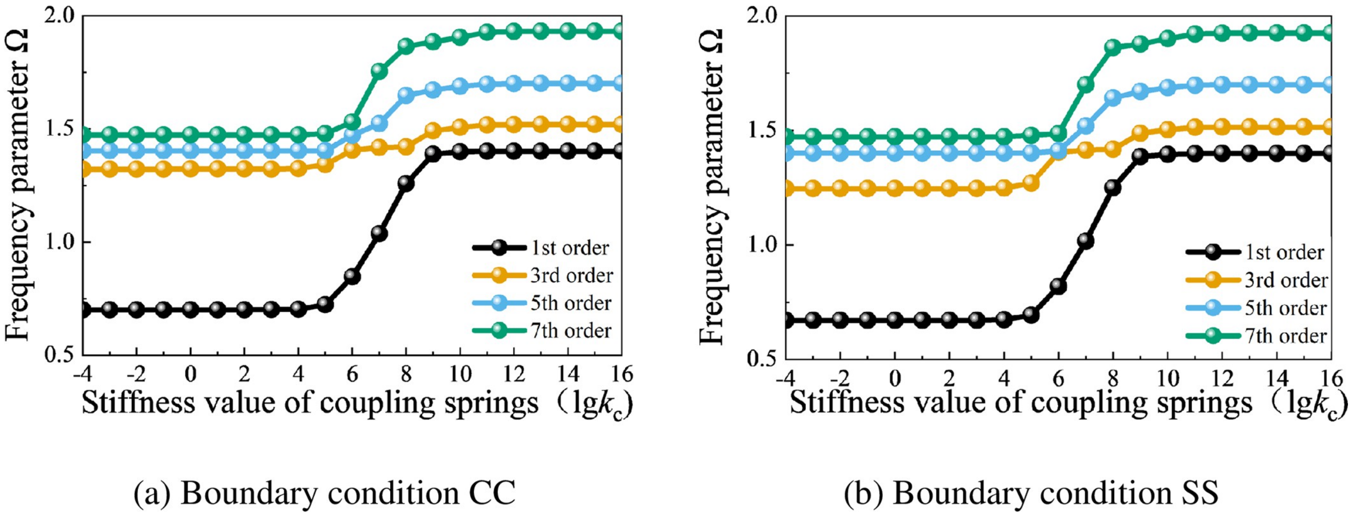

Fig 7. Variation curves of the frequency parameter Ω of the closed composite cylindrical shell under different shell internal coupling spring stiffness values. (a) Boundary condition CC. (b) Boundary condition SS.

https://doi.org/10.1371/journal.pone.0299586.g007

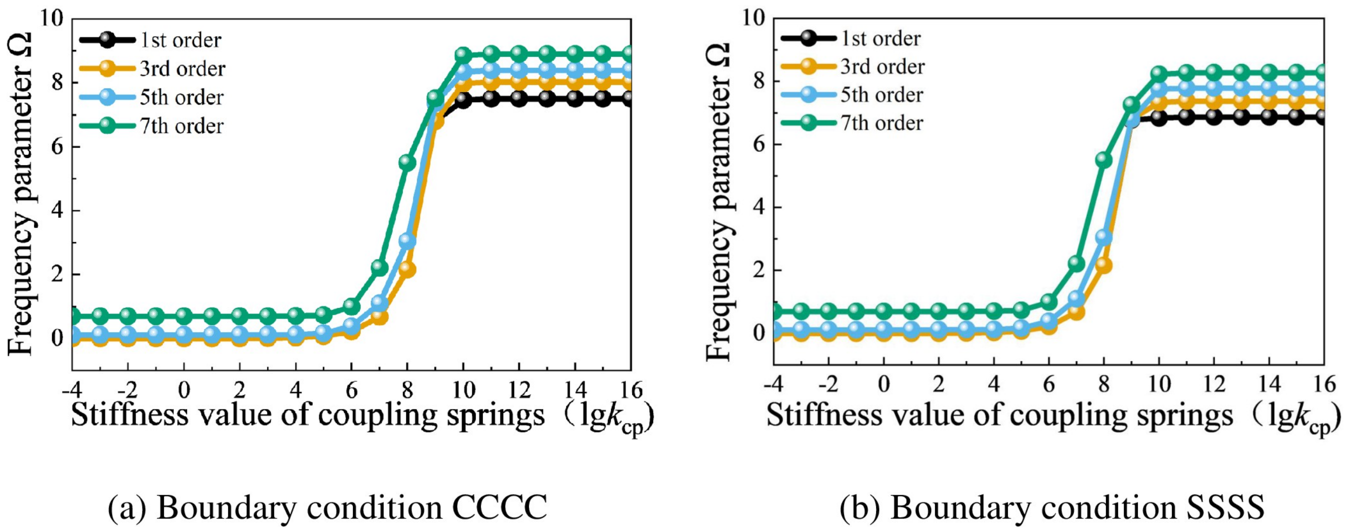

Fig 8. Variation curves of the frequency parameter Ω of the open composite stiffened conical shell under different coupling springs stiffness values between the shell and beams. (a) Boundary condition CCCC. (b) Boundary condition SSSS.

https://doi.org/10.1371/journal.pone.0299586.g008

increase with the increase of k_c, and they stabilize when k_c increases to 10^{10}. At this point, rigid coupling simulation can be achieved. To ensure the correctness of the calculation results, the shell internal coupling spring stiffness value k_c is set to 10^{16} in subsequent cases.

Next, taking an open composite stiffened conical shell as an example, the influence of coupling conditions between the shell and beam structures in the composite laminated rotationally stiffened shell structure will be studied. The case selects material parameters and geometric parameters with values identical to those in Table 3. Fig 8 provides the variation curves of frequency parameters with the stiffness values of coupling springs under two different boundary conditions: CCCC (clamped-clamped-clamped-clamped) and SSSS (simply supported-simply supported-simply supported-simply supported). From Fig 8, it can be observed that the influence of the coupling spring stiffness value k_{cp} on the frequency parameter Ω is similar to that of the shell internal coupling spring stiffness value k_c. When the stiffness value k_{cp} is less than 10^4, it has a minimal impact on the frequency parameter Ω. When the stiffness value k_{cp} increases to 10^{10}, it can achieve rigid coupling of the composite rotationally shell with the laminated curved beam at the coupling boundary.

3.2 Free vibration analysis

This section investigates the influence of various parameters on the vibration characteristics of stiffened shells. We begin by analyzing the impact of relevant parameters of composite rotationally shell structures on the vibration characteristics of stiffened shells. Taking the example of an open stiffened cylindrical shell, Table 5 provides the frequency parameters Ω of the open composite stiffened cylindrical shell under different shell boundary conditions, thicknesses, and rotation angles. In this case, the stiffened shell includes two laminated beams as stiffener, with stiffener 1 located at $2L_s/3$ and stiffener 2 at $L_s/3$. The material of the laminated shell is glass epoxy resin, and the material of the laminated beams is graphite epoxy resin, with ply angles of [60˚/0˚/60˚]. The fixed geometric parameters for this case are $R_s = R_{b1} = 1$m, $L_s = 3$m, $b_1 = b_2 = 0.03$m, $h_1 = h_2 = 0.06$m. From Table 5, it can be observed that the boundary conditions, thickness, and the rotation angle of the stiffened shell in open composite stiffened

Table 5. Frequency parameters Ω of the open composite stiffened cylindrical shell under different shell boundary conditions and parameter condition.

Boundary condition	h_s/R_s	ϑ	Modal order							
			1	2	3	4	5	6	7	8
CCCC	0.05	90˚	1.673	1.915	2.152	2.158	2.880	2.895	3.749	3.835
		180˚	1.112	1.180	1.473	1.503	1.680	1.745	1.907	2.006
		270˚	0.992	1.003	1.262	1.327	1.379	1.380	1.601	1.610
	0.01	90˚	0.703	0.879	0.937	0.988	1.468	1.487	1.751	1.799
		180˚	0.481	0.484	0.636	0.656	0.716	0.780	0.866	0.917
		270˚	0.418	0.428	0.535	0.560	0.588	0.595	0.698	0.718
CCSS	0.05	90˚	1.387	1.789	1.906	1.956	2.708	2.710	3.139	3.205
		180˚	1.094	1.094	1.435	1.487	1.549	1.666	1.824	1.941
		270˚	0.966	1.000	1.194	1.296	1.363	1.379	1.539	1.548
	0.01	90˚	0.611	0.779	0.841	0.850	1.421	1.431	1.445	1.480
		180˚	0.449	0.483	0.626	0.635	0.639	0.758	0.847	0.849
		270˚	0.415	0.417	0.524	0.530	0.585	0.589	0.672	0.689
SSSS	0.05	90˚	1.383	1.786	1.905	1.955	2.707	2.710	3.138	3.203
		180˚	1.091	1.092	1.431	1.484	1.548	1.664	1.823	1.940
		270˚	0.962	0.997	1.192	1.293	1.359	1.375	1.537	1.545
	0.01	90˚	0.610	0.779	0.841	0.849	1.420	1.431	1.445	1.480
		180˚	0.448	0.483	0.626	0.635	0.639	0.758	0.847	0.849
		270˚	0.415	0.417	0.524	0.530	0.585	0.589	0.672	0.689
$E^2E^2E^2E^2$	0.05	90˚	0.143	0.145	0.150	0.214	0.483	0.522	0.659	0.788
		180˚	0.109	0.121	0.123	0.158	0.239	0.448	0.486	0.525
		270˚	0.091	0.111	0.112	0.131	0.167	0.236	0.382	0.391
	0.01	90˚	0.125	0.141	0.165	0.172	0.225	0.269	0.274	0.430
		180˚	0.100	0.123	0.135	0.147	0.151	0.199	0.245	0.248
		270˚	0.095	0.102	0.123	0.124	0.140	0.150	0.176	0.235

https://doi.org/10.1371/journal.pone.0299586.t005

cylindrical shells all have an impact on their frequency parameter Ω. As thickness h_s is varied in this case, the dimensionless natural frequency parameter is defined as $\Omega = \omega/R_s\sqrt{100\rho_s/E_1}$.

Next, the influence of boundary conditions, thickness-to-radius ratio (h_s/R_s), and rotation angle (ϑ) on the frequency parameter Ω of composite rotationally stiffened shell is presented, taking the example of the stiffened cylindrical shell from Table 5. Fig 9 illustrates the variation curves of frequency parameter Ω with the thickness ratio h_s/R_s or rotation angle ϑ under different boundary conditions. The frequency parameter Ω of the open composite stiffened cylindrical shell increases with the increase of thickness ratio h_s/R_s and decreases with the increase of the rotation angle ϑ. Simultaneously, based on the positions of the curves corresponding to different boundary conditions in Fig 9, it can be observed that the frequency parameter Ω increases with the increase of spring stiffness.

Table 6 shows the influence of layer angles, conical shell length, and apex angle variation on the frequency parameter Ω of closed composite laminated stiffened conical shells. In this example, the stiffened shell has two laminated curved beams as stiffener, with the first one located at $L_s/2$ and the second one at $2L_s/3$. The materials of laminated shell and curved beams are both graphite epoxy resin. The fixed geometric parameters in this example are: R_1 = 2m, h_s = 0.1m, $b_1 = b_2$ = 0.1m, $h_1 = h_2$ = 0.2m, $R_2 = R_1+L_S \sin\varphi$, $R_{b1} = R_1+(L_S/2)\sin\varphi$,$R_{b2} = R_1+(2L_S/3)\sin\varphi$,$\vartheta$ = 360˚. From Table 6, it can be observed that the layer angles, the length ratio, and the apex angle of the stiffened shell in closed composite laminated stiffened conical shells also

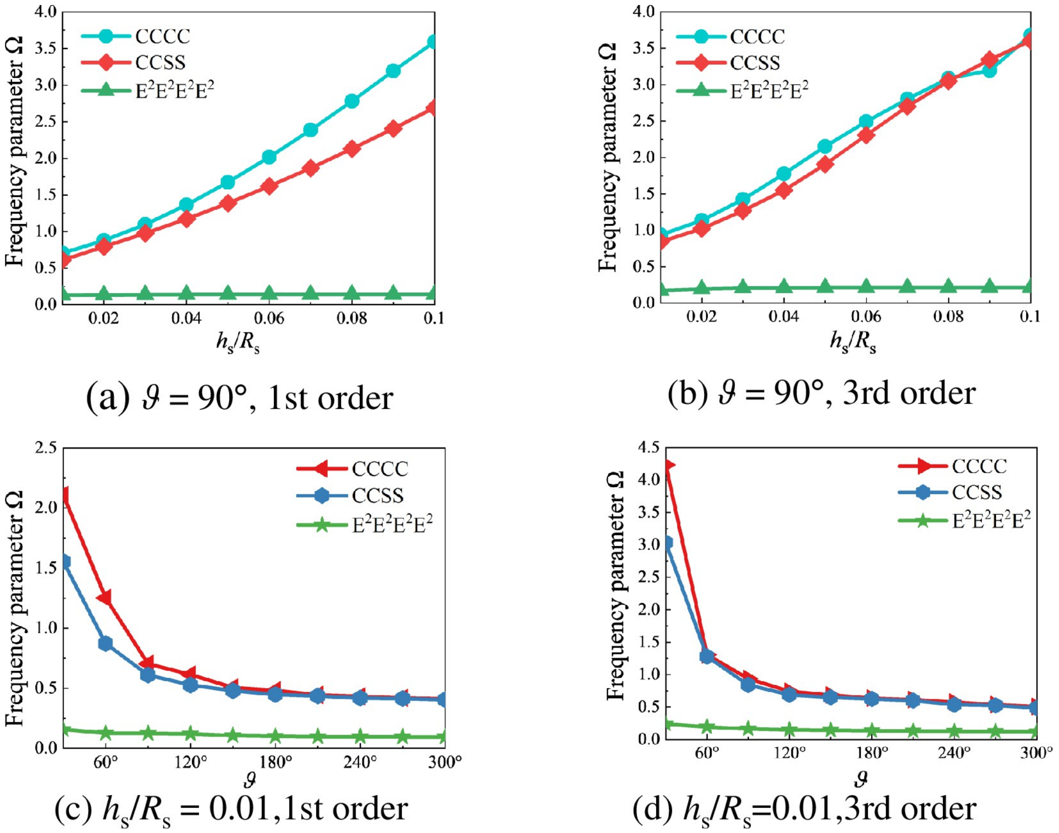

Fig 9. Variation curves of frequency parameter Ω of the open composite stiffened cylindrical shell with the thickness ratio h_s/R_s or rotation angle ϑ under different boundary conditions. (a) ϑ = 90˚, 1st order. (b) ϑ = 90˚, 3rd order. (c) h_s/R_s = 0.01, 1st order. (d) h_s/R_s = 0.01, 3rd order.

https://doi.org/10.1371/journal.pone.0299586.g009

affect their frequency parameter Ω. To visually demonstrate the impact, Fig 10 provides variation curves of the first few mode frequency parameters Ω with the length ratio L_s/R_1 under different layer angles and apex angle φ. It is evident from the curves that the frequency parameter Ω of the closed composite laminated stiffened conical shell decreases with the increase of the length ratio L_s/R_1. Based on the positions of the curves corresponding to different apex angles φ in the figure, it can be concluded that the frequency parameter Ω of the same modal order decreases with the increase of the apex angle φ.

Additionally, the influence of parameter conditions of composite laminated curved beams on the vibration characteristics of composite laminated stiffened shells was analyzed. Table 7 presents the frequency parameters Ω of the first eight modes of composite laminated stiffened cylindrical shells with different numbers of stiffeners. The maximum value of the number of stiffeners (n) is 3, where the first stiffener is located at $L_s/2$, the second one at $L_s/3$, and the third one at $2L_s/3$. The geometric parameters of the composite laminated stiffened cylindrical shell in this example are: $R_s = R_{b1} = R_{b2} = R_{b3} = 1.5$m, $L_s = 3.6$m, $b_1 = b_2 = b_3 = 0.08$m, $h_1 = h_2 = h_3 = 0.1$m. The shell material is set as glass fiber resin, and the stiffener material is set as steel, with layer angles set as [0˚/90˚]. From Table 7, it can be observed that, compared to the cylindrical shell structure without stiffener, the frequency parameters Ω of most modes of the stiffened cylindrical shell with one stiffener (n = 1) undergo significant changes and tend to decrease with an increase in the number of stiffeners (n).

Table 6. Frequency parameter Ω of closed composite laminated stiffened conical shells under different parameters.

Layer angle	L_s/R_1	φ	Modal order							
			1	2	3	4	5	6	7	8
[0˚/45˚]	1.5	15˚	1.726	1.936	1.936	1.941	1.942	1.958	1.958	1.965
		30˚	1.692	1.814	1.814	1.837	1.838	1.843	1.843	1.861
		45˚	1.632	1.691	1.691	1.719	1.719	1.724	1.724	1.725
	3	15˚	0.872	0.872	0.890	0.890	0.924	0.926	0.927	0.968
		30˚	0.785	0.786	0.808	0.808	0.814	0.816	0.871	0.871
		45˚	0.691	0.692	0.703	0.703	0.716	0.718	0.742	0.742
[0˚/90˚]	1.5	15˚	1.786	1.787	1.807	1.808	1.855	1.855	1.914	1.915
		30˚	1.703	1.703	1.719	1.720	1.758	1.758	1.805	1.806
		45˚	1.600	1.600	1.624	1.624	1.630	1.630	1.703	1.704
	3	15˚	0.794	0.795	0.828	0.828	0.864	0.866	0.982	0.982
		30˚	0.712	0.716	0.716	0.753	0.754	0.757	0.758	0.864
		45˚	0.632	0.632	0.662	0.662	0.663	0.664	0.722	0.751
[-90˚/0˚/90˚]	1.5	15˚	1.531	1.531	1.551	1.551	1.747	1.748	1.807	1.808
		30˚	1.417	1.417	1.441	1.441	1.590	1.590	1.661	1.661
		45˚	1.293	1.293	1.294	1.294	1.456	1.456	1.456	1.457
	3	15˚	0.778	0.778	0.888	0.889	0.916	0.916	0.965	0.965
		30˚	0.691	0.691	0.742	0.743	0.801	0.801	0.830	0.830
		45˚	0.589	0.589	0.629	0.629	0.693	0.694	0.701	0.701

https://doi.org/10.1371/journal.pone.0299586.t006

Table 8 presents the frequency parameters Ω of the first eight modes of two types of composite laminated stiffened conical shells with different numbers of stiffener. The maximum value of the number of stiffener (n) is 3, where the first stiffener is located at $L_s/2$, the second one at $L_s/3$, and the third one at $2L_s/3$. The geometric parameters of the composite laminated stiffened conical shell in this example are: R_1 = 1.5m, L_s = 3.6m,φ = 15˚,$b_1 = b_2 = b_3$ = 0.08m,$h_1 = h_2 = h_3$ = 0.1m, $R_2 = R_1 + L_S \sin\varphi$, $R_{b1} = R_1 + (L_S/2) \sin\varphi$, $R_{b2} = R_1 + (L_S/3) \sin\varphi$, $R_{b3} = R_1 + (2L_S/3) \sin\varphi$. The shell material is set as glass fiber resin, and the stiffener material is set as steel, with layer angles set as [0˚/90˚]. As shown in Table 8, the frequency parameters Ω of the stiffened conical shell tend to increase with an increase in the number of stiffeners (n).

Taking the closed stiffened cylindrical shell as an example, Fig 11 illustrates the mode shapes corresponding to the first and third frequency parameters under different numbers of stiffener. This provides a more intuitive representation of the influence of stiffener on the rotationally shell structure. The specific dimensions and material parameters of this example correspond to those in Table 7. It is evident that the mode shape contours at the location of the stiffener undergo noticeable distortion, indicating that the coupled structure of laminated shell and laminated curved beams, as modeled in the composite rotationally stiffened shell, has been successfully captured.

Fig 12 shows the variation curves of frequency parameters Ω with the width b_n of the laminated curved beams for both open stiffened cylindrical shells and conical shells at the different thickness-to-width ratio. In this case, the number of stiffener n is 2, with stiffener 1 located at $R_p/3$ and stiffener 2 located at $2R_p/3$. The geometric parameters of the composite stiffened cylindrical shell are: $R_s = R_{b1} = R_{b2}$ = 1m, L_s = 3m,h_s = 0.01m,ϑ = 120˚; and for the stiffened conical shell: R_1 = 1m, L_s = 3m, h_s = 0.01m, φ = 30˚, ϑ = 120˚, $R_2 = R_1 + L_S \sin\varphi$,$R_{b1} = R_1 + (L_S/2) \sin\varphi$ $R_{b2} = R_1 + (L_S/3) \sin\varphi$, $R_{b3} = R_1 + (2L_S/3) \sin\varphi$. The boundary conditions are $E^1E^1E^1E^1$, and the materials of the shell structure and stiffeners are set as graphite fiber-stiffened resin, with layer angles set to [-90˚/0˚/90˚]. From Fig 12, it can be observed that the frequency parameters

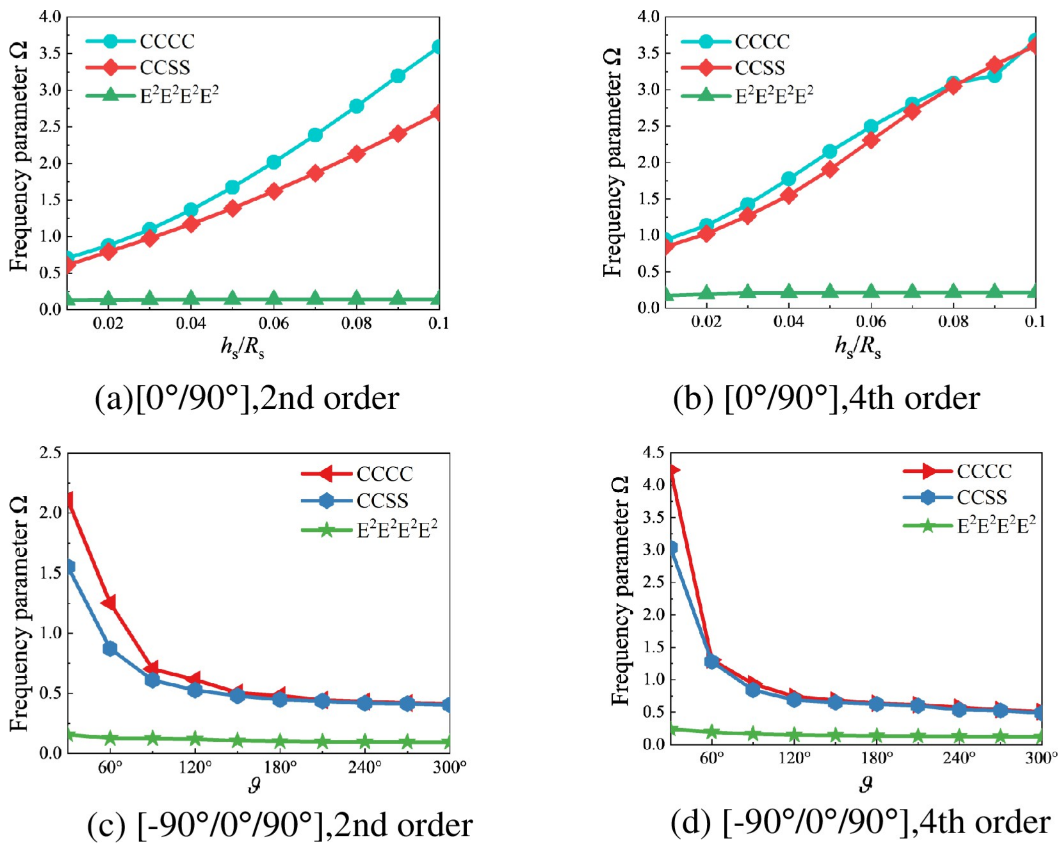

Fig 10. variation curves of the first few mode frequency parameters Ω of the closed composite laminated stiffened conical shell with the length ratio L_s/R_1 under different layer angles and apex angle *φ*. (a) [0˚/90˚],2nd order. (b) [0˚/90˚], 4th order. (c) [-90˚/0˚/90˚],2nd order. (d) [-90˚/0˚/90˚],4th order.

https://doi.org/10.1371/journal.pone.0299586.g010

Ω increase with the increasing thickness h_n of the laminated curved beams at the different thickness-to-width ratio. In this case, as the thickness of the laminated curved beams increases, the width also increases, indicating a positive correlation between the thickness and width of the laminated curved beams with the frequency parameters Ω of the composite rotationally stiffened shell.

Table 7. Frequency parameter Ω of composite laminated stiffened shells under different number of stiffeners.

Number of stiffeners *n*	Modal order							
	1	2	3	4	5	6	7	8
	Open stiffened cylindrical shell: ϑ = 90˚, h_s = 0.03m, SSSS							
$n = 0$	3.659	4.820	5.005	6.985	7.281	7.367	9.318	9.372
$n = 1$	3.817	5.352	8.968	9.097	9.167	9.313	9.483	10.541
$n = 2$	3.438	5.371	5.851	6.223	9.085	9.133	9.190	9.427
$n = 3$	3.259	5.092	5.376	5.868	6.350	6.605	9.136	9.176
	Closed stiffened cylindrical shell: ϑ = 360˚, h_s = 0.08m,SS							
$n = 0$	2.250	2.255	2.361	2.361	2.763	2.778	3.403	3.403
$n = 1$	1.159	1.159	1.327	1.328	1.628	1.628	1.860	1.863
$n = 2$	1.039	1.039	1.257	1.259	1.386	1.386	1.801	1.810
$n = 3$	0.988	0.988	1.225	1.228	1.265	1.265	1.769	1.778

https://doi.org/10.1371/journal.pone.0299586.t007

Table 8. Frequency parameter Ω of composite laminated stiffened conical shell under different number of stiffeners.

Number of stiffeners n	Modal order							
	1	2	3	4	5	6	7	8
	Open stiffened conical shell: $\vartheta = 90^\circ$, $h_s = 0.03$m, SSSS							
$n = 0$	3.603	3.866	5.275	5.538	6.051	6.345	7.290	7.291
$n = 1$	6.110	6.164	7.276	7.294	7.454	7.582	9.267	9.385
$n = 2$	6.117	6.171	7.440	7.580	9.291	9.432	10.280	10.290
$n = 3$	8.713	8.713	9.497	9.521	10.448	10.478	10.711	10.748
	Closed stiffened conical shell: $\vartheta = 360^\circ$, $h_s = 0.08$m, SS							
$n = 0$	1.725	1.873	1.874	1.976	2.036	2.364	2.458	2.506
$n = 1$	2.779	2.788	2.808	2.815	2.844	2.845	2.869	2.869
$n = 2$	3.404	3.415	3.513	3.517	3.695	3.696	3.880	3.880
$n = 3$	4.419	4.419	4.480	4.480	4.517	4.518	4.555	4.572

https://doi.org/10.1371/journal.pone.0299586.t008

The stiffened shell model established in this paper can also be used to study the vibration characteristics of isotropic rotationally stiffened shells. Taking the closed stiffened cylindrical shell and closed stiffened conical shell as examples, Table 9 provides the first eight natural frequency parameters Ω of isotropic rotationally stiffened shells under different boundary conditions. A comparison is made with finite element results and results from the literature. In this case, the number of stiffeners n is set to 1, and the stiffener is located at $L_s/3$. The geometric parameters of the isotropic stiffened cylindrical shell are: $R_s = 2$m, $L_s = 5$m; and for the stiffened conical shell: $R_1 = 1$m, $L_s = 10$m, $\varphi = 30^\circ$. The material parameters of the shell structure and stiffeners are set to $E_1 = E_2 = 185$GPa, $\mu_{12} = 0.3$, $G_{12} = G_{13} = G_{23} = 71.2$GPa, $\rho_s = 1600$ kg/m^3, representing an isotropic material. The frequency parameters of the stiffened cylindrical shell $\Omega = \omega/h_s\sqrt{\rho_s/E_1}$, while for the stiffened conical shell, $\Omega = \omega R_1\sqrt{\rho_s(1-\mu_{12}{}^2)/E_1}$. It can be observed that the results obtained by the proposed method in Table 9 are in good agreement with those obtained by other methods.

In order to validate the accuracy of the model of composite rotationally stiffened shells established in this paper, modal tests were conducted using a closed stiffened cylindrical shell with free boundary condition as an example. The experimental results were then compared with the results obtained through present method. The modal test of the closed stiffened cylindrical shell was conducted using the frequency response function method. The test instruments include the LC02 force hammer, 3A105 force sensor, DH5857-1 charge adjuster, 1A116E acceleration sensor, and DH5922D dynamic signal test and analysis system. Single point pickup method is adopted when collecting data, the position of the sensor remains unchanged, and hammer is used to beat the intersection point of the grid drawn before. After hitting all the test points set, the peak position of the frequency response curve drawn is determined in the test software according to the collected data, which is the natural frequency of the test stiffened plate; by calculating the mode in the test software, the relevant mode can be obtained.

The dimensional parameters of the closed stiffened cylindrical shell with free boundary condition are as follows: $R_s = 0.102$m, $L_s = 0.355$m, $h_s = 0.002$m, $b_1 = 0.005$m, $h_1 = 0.005$m, $\vartheta = 360^\circ$. The material of the laminated shell is 304 stainless steel, and there is a single laminated beam located at $L_s/2$, also made of 304 stainless steel. The specific material parameters are: $E = 194$ GPa, $\mu = 0.3$, and $\rho_s = 7930$kg/m^3. Additionally, two through-holes with a diameter of 10mm are symmetrically punched on the shell structure to allow the passage of elastic cords.

Fig 13 illustrates the layout of the closed stiffened cylindrical shell and the experimental setup for the modal test. Because the laminated shell is suspended by elastic cords, the

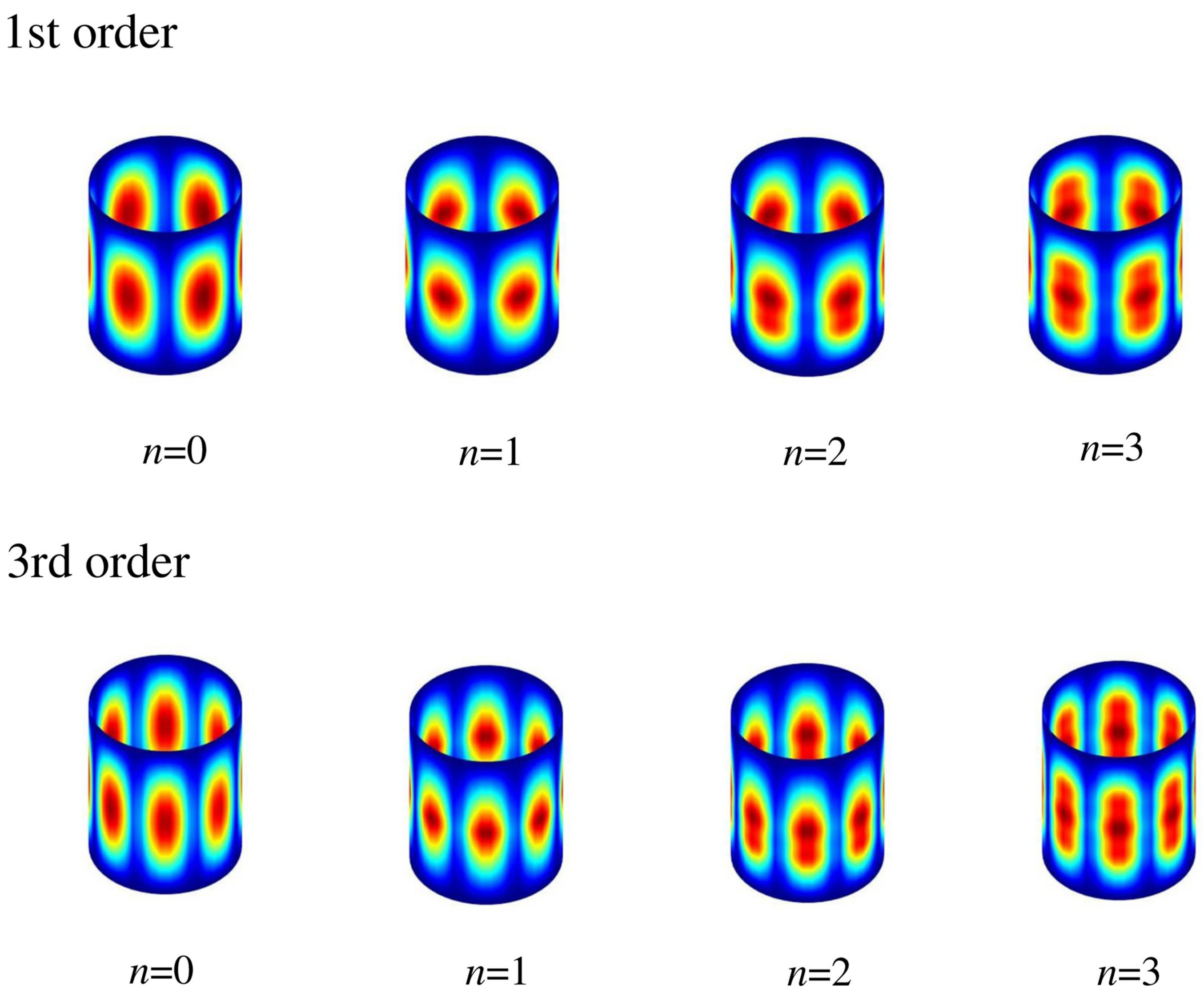

Fig 11. Modal diagrams of composite closed stiffened cylindrical shells under different number of stiffeners. 1st order: $n = 0$, $n = 1$, $n = 2$, $n = 3$; 3rd order: $n = 0$, $n = 1$, $n = 2$, $n = 3$.

https://doi.org/10.1371/journal.pone.0299586.g011

laminated shell is in free boundary condition. The acceleration sensor and the force hammer are connected to the dynamic signal test and analysis system. In this case, the stiffened cylindrical shell needs to be divided into elements: 8 divisions in the θ direction and 5 divisions in the x direction, totaling 48 measurement points. Accelerometers are placed at the measurement point 10. The data is collected by hammering at 48 different points. According to the collected data, the frequency response curve can be obtained in the test software, and the peak value of the frequency response curve is the natural frequency of the test stiffened shell. Fig 14 shows the natural frequencies and mode shapes of the third, fifth, and ninth modes obtained from experimental tests and the present method. From the content of Fig 14, it can be observed that the maximum error between the natural frequency of experimental test results and the natural frequency of calculated results of this paper is 2.28%, which is within an acceptable range. At the same time, the mode shapes obtained from the two methods are also in good agreement, further confirming the correctness of the established model. The experimental

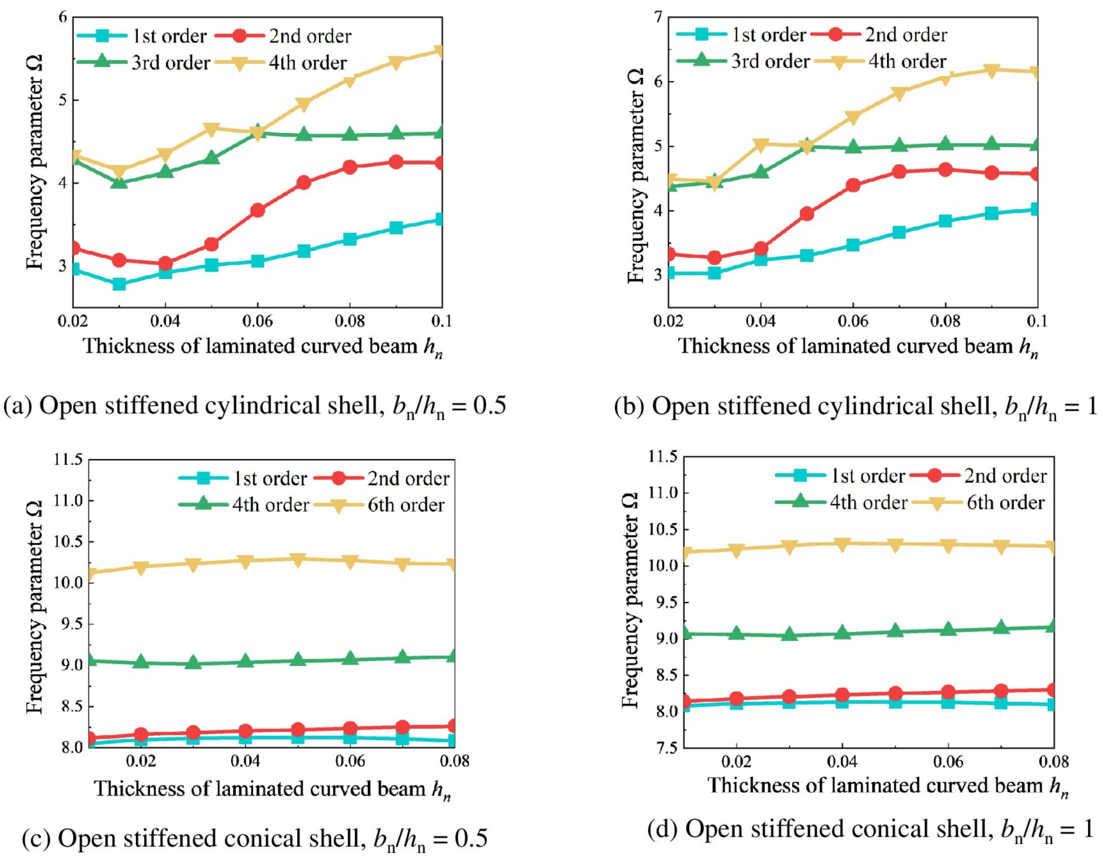

Fig 12. variation curves of the frequency parameters Ω of the composite laminated rotationally stiffened shell with the width of the laminated curved beam under different thickness-to-width ratio. (a) Open stiffened cylindrical shell, $b_n/h_n = 0.5$. (b) Open stiffened cylindrical shell, $b_n/h_n = 1$. (c) Open stiffened conical shell,$b_n/h_n = 0.5$. (d) Open stiffened conical shell,$b_n/h_n = 1$.

https://doi.org/10.1371/journal.pone.0299586.g012

error is caused by many reasons. First of all, the free boundary conditions of the stiffened shell cannot be fully simulated. Then, the material parameters used in the numerical calculation of the stiffened shell deviate from the actual material parameters of the workpiece, and the workpiece cannot be completely ideal isotropic material. In addition, the accuracy deviation of the

Table 9. Frequency parameter Ω of isotropic rotationally stiffened shells under different boundary conditions.

Boundary condition	Method	Modal order							
		1	2	3	4	5	6	7	8
		Closed stiffened cylindrical shell: $\vartheta = 360^\circ$, $h_s = 0.07$m, $b_1 = 0.1$m, $h_1 = 0.2$m							
CF	Present method	0.049	0.049	0.103	0.103	0.159	0.159	0.194	0.194
	FEM	0.051	0.051	0.110	0.110	0.157	0.157	0.193	0.193
CC	Present method	0.188	0.188	0.195	0.195	0.249	0.249	0.262	0.262
	FEM	0.186	0.186	0.196	0.196	0.247	0.247	0.253	0.253
		Closed stiffened conical shell: $\vartheta = 360^\circ$, $h_s = 0.05$m, $b_1 = 0.06$m, $h_1 = 0.08$m							
CF	Present method	0.036	0.036	0.102	0.102	0.147	0.147	0.166	0.166
	Reference[22]	0.036	0.036	0.101	0.101	0.146	0.146	0.165	0.165
CC	Present method	0.102	0.102	0.132	0.132	0.163	0.163	0.179	0.179
	Reference [22]	0.102	0.102	0.131	0.131	0.161	0.161	0.178	0.178

https://doi.org/10.1371/journal.pone.0299586.t009

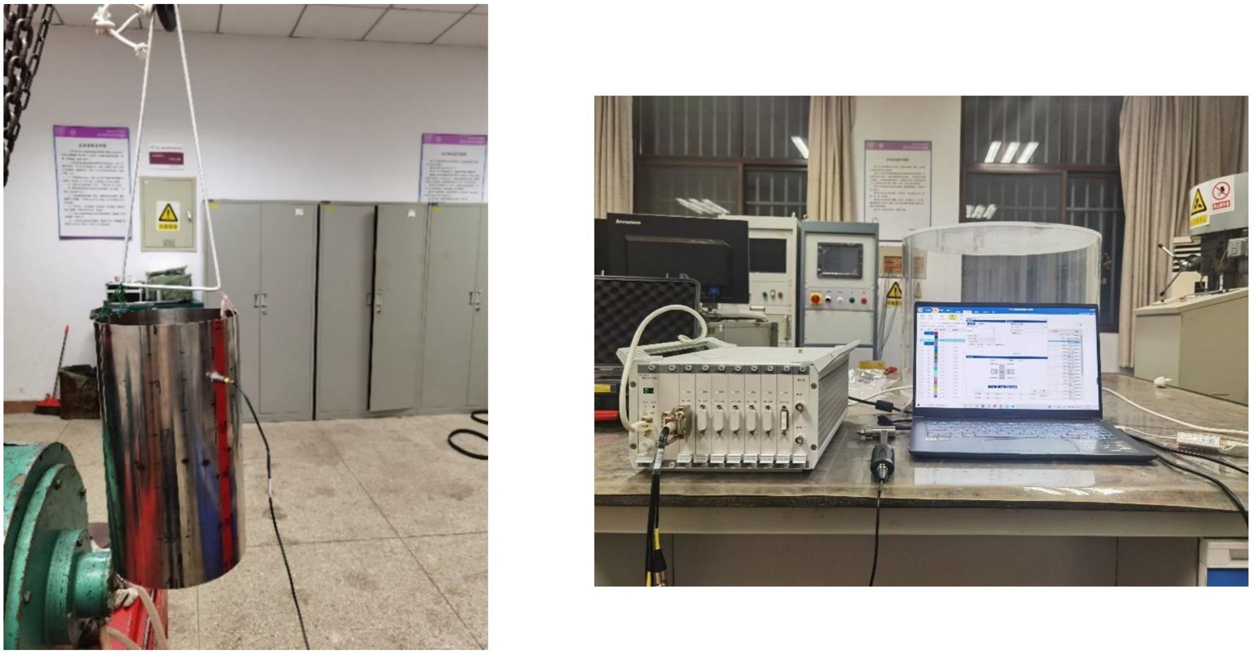

Fig 13. Layout of the closed stiffened cylindrical shell and the experimental setup.

https://doi.org/10.1371/journal.pone.0299586.g013

force sensor and the acceleration sensor, and the human error of the experimenter in the process of hammering will cause the error in experimental data.

4 Conclusions

This paper established a unified analytical model for the vibration characteristics of composite stiffened cylindrical shell, specifically stiffened cylindrical and conical shells, based on the improved Fourier series method and the Rayleigh-Ritz method. The total energy functional of composite stiffened cylindrical shell is obtained and solved using these methods. The study investigates the free vibration characteristics of composite stiffened cylindrical shell, leading to the following key conclusions:

1. When the truncation values of the displacement admissible functions of laminated shells and laminated curved beams are set to $M_s \times N_s = 18 \times 18$ and $M_b = 25$, the natural frequencies obtained by the unified analytical model for the vibration characteristics of composite stiffened cylindrical shell constructed by present method show a maximum error of 3.77% compared to the finite element method. Moreover, the stiffness values of each spring can generally converge around 10^{10}.
2. The natural frequencies of composite stiffened cylindrical shell increase with the growth of the thickness ratio, increase with the rise of boundary spring stiffness values, decrease with the increase of the length ratio. For the case of stiffened conical shell, the natural frequencies decrease with the increase of the cone apex angle.
3. The natural frequencies of composite stiffened cylindrical shell increase with the increase of the thickness and width of laminated beams. For stiffened cylindrical shells, the natural frequencies decrease with the increase in the number of stiffeners, while for stiffened conical shells, the natural frequencies increase with the increase in the number of stiffeners.

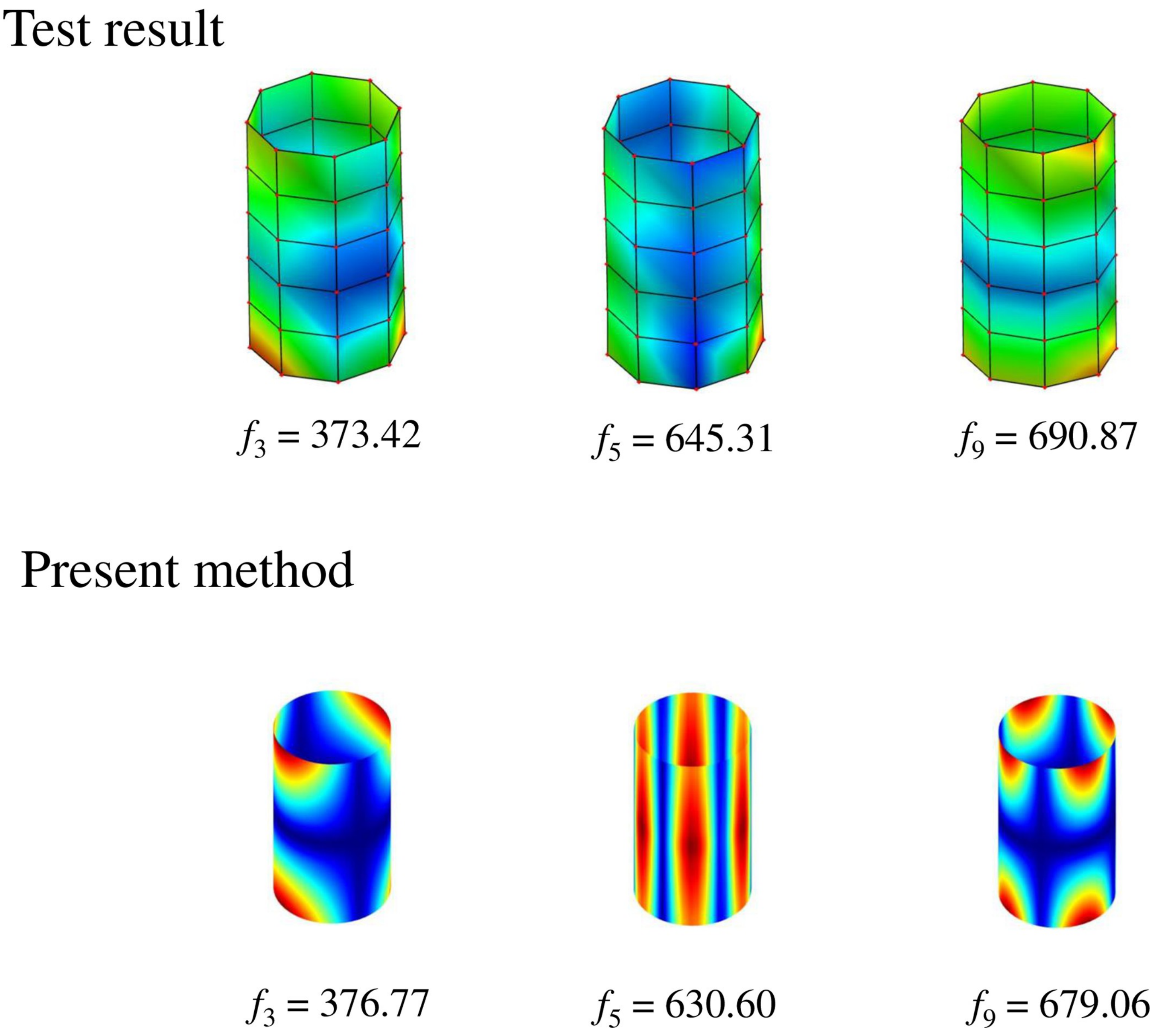

Fig 14. Natural frequencies and mode shapes of the stiffened cylindrical shell obtained by the test and the present method. Test result: f_3 = 373.42, f_5 = 645.31, f_9 = 690.87; Present method: f_3 = 376.77, f_5 = 630.60, f_9 = 679.06.

https://doi.org/10.1371/journal.pone.0299586.g014

Supporting information

S1 File.
(ZIP)

Author Contributions

Conceptualization: Yiqun Ding.

Data curation: Changrun Yang.

Formal analysis: Tianlin Cheng.

Funding acquisition: Dong Shi.

Investigation: Dong Shi.

Methodology: Dong Shi.

Project administration: Dong Shi.

Resources: Dong Shi.

Software: hong zhang.

Supervision: hong zhang.

Validation: hong zhang.

Visualization: hong zhang.

Writing – original draft: hong zhang.

Writing – review & editing: hong zhang.

References

1. Thein W, Circular Symmetric Vibrations of Ring-Stiffened Cylindrical Shells. Journal of the Society for Industrial and Applied Mathematics, 1964. 12(3):p. 649–662.
2. Thein W, Flexural vibrations of ring-stiffened cylindrical shells. Academic Press, 1966. 3(3): p. 242–251. https://doi.org/10.1016/0022-460X(66)90093-9
3. Weingarten V I, Free vibrations of ring-stiffened conical shells. Aiaa Journal, 1965. 3(8): p. 1475–1481.
4. Al-Najafi A M, Warburton G B, Free vibration of ring-stiffened cylindrical shells. Academic Press, 1970. 13(1): p. 9–25.
5. Stanley A J, Ganesan N, Free vibration characteristics of stiffened cylindrical shells. Computers and Structures, 1997. 65(1): p. 33–45.
6. Sharma C B, Johns D J, Vibration characteristics of a clamped-free and clamped-ring-stiffened circular cylindrical shell. Academic Press, 1971. 14(4): p. 459–474. https://doi.org/10.1016/0022-460X(71)90575-X
7. Qu Y, Chen Y, Long X, A modified variational approach for vibration analysis of ring-stiffened conical–cylindrical shell combinations. European Journal of Mechanics, a, Solids, 2013. 37: p. 200–215.
8. Chen M, Xie K, Jia W, Free and forced vibration of ring-stiffened conical–cylindrical shells with arbitrary boundary conditions. Ocean Engineering, 2015. 108: p. 241–256.
9. Shen H, Xiang Y, Nonlinear vibration of nanotube-reinforced composite cylindrical shells in thermal environments. Computer Methods in Applied Mechanics and Engineering, 2012. 213–216: p. 196–205.
10. Li D, Qing G, Liu Y, A layerwise/solid-element method for the composite stiffened laminated cylindrical shell structures. Composite Structures, 2013. 98: p. 215–227.
11. Sahoo S, Laminated composite stiffened shallow spherical panels with cutouts under free vibration–A finite element approach. Engineering Science and Technology, an International Journal, 2014. 17(4): p. 247–259.
12. Thomas B, Roy T, Vibration analysis of functionally graded carbon nanotube-reinforced composite shell structures. Acta Mechanica, 2016. 227(2): p. 581–599.
13. Rout M, Hota S S, Karmakar A, Free vibration characteristics of delaminated composite pretwisted stiffened cylindrical shell. Proceedings of the Institution of Mechanical Engineers, Part C: Journal of Mechanical Engineering Science, 2018. 232(4): p. 595–611.
14. Guo C, Tao L, Qingshan W, Qin B, Wen S, Ailun W, Spectral-Tchebychev technique for the free vibration analysis of composite laminated stepped and stiffened cylindrical shells with arbitrary boundary conditions. Composite Structures, 2021. 272: p. 114193.
15. Abedini B A, Kiani Y, Free vibration analysis of functionally graded cylindrical shells reinforced with graphene platelets. Composite Structures, 2021. 276: p. 114546.
16. Dong Y, Gao Y, Zhu Q, Moradi Z, Safa M, TE-GDQE implementation to investigate the vibration of FG composite conical shells considering a frequency controller solid ring. Engineering Analysis with Boundary Elements, 2022. 138: p. 95–107.

17. Zhang H., Shi D., Zha S., Wang Q., Vibro-acoustic analysis of the thin laminated rectangular plate-cavity coupling system. Composite Structures, 2018. 189: p. 570–585.

18. Shi D., Zha S., Zhang H., Wang Q., Free vibration analysis of the unified functionally graded shallow shell with general boundary conditions. Shock and Vibration, 2017. 2017: p. 7025190.

19. Shi D., Zhang H., Wang Q., Zha S., Free and Forced Vibration of the Moderately Thick Laminated Composite Rectangular Plate on Various Elastic Winkler and Pasternak Foundations. Shock and Vibration, 2017. 2017: p. 7820130.

20. Zhang H., Shi D., Zha S., Wang Q., Parameterization study on the moderately thick laminated rectangular plate-cavity coupling system with uniform or non-uniform boundary conditions. Composite Structures, 2018. 194: p. 537–554.

21. Ramkumar K. and Ganesan N., Vibration and damping of composite sandwich box column with viscoelastic/electrorheological fluid core and performance comparison. Materials & Design, 2009. 30(8): p. 2981–2994.

22. Bagheri H, Kiani Y, Eslami M R, Free vibration of conical shells with intermediate ring support. Aerospace Science and Technology, 2017. 69: p. 321–332.

23. Rout M, Bandyopadhyay T, Karmakar A, Free vibration analysis of pretwisted delaminated composite stiffened shallow shells: a finite element approach. Journal of Reinforced Plastics and Composites, 2017. 36(8): p. 619–636.

24. Zarei M, Rahimi G H, Hemmatnezhad M, Free vibrational characteristics of grid-stiffened truncated composite conical shells. Aerospace Science and Technology, 2020. 99: p. 105717.
